# Identification of the Antifungal Metabolite Chaetoglobosin P From *Discosia rubi* Using a *Cryptococcus neoformans* Inhibition Assay: Insights Into Mode of Action and Biosynthesis

**DOI:** 10.3389/fmicb.2020.01766

**Published:** 2020-07-28

**Authors:** Bruno Perlatti, Connie B. Nichols, Nan Lan, Philipp Wiemann, Colin J. B. Harvey, J. Andrew Alspaugh, Gerald F. Bills

**Affiliations:** ^1^Texas Therapeutics Institute, The Brown Foundation Institute of Molecular Medicine, University of Texas Health Science Center at Houston, Houston, TX, Untied States; ^2^Departments of Medicine and Molecular Genetics and Microbiology, Duke University Medical Center, Durham, NC, United States; ^3^Hexagon Bio, Menlo Park, CA, United States

**Keywords:** actin, β-methyltryptophan, cytochalasins, *Stenocarpella macrospora*, twinfilin-1, Xylariales

## Abstract

*Cryptococcus neoformans* is an important human pathogen with limited options for treatments. We have interrogated extracts from fungal fermentations to find *Cryptococcus*-inhibiting natural products using assays for growth inhibition, differential thermosensitivity, and synergy with existing antifungal drugs. Extracts from fermentations of strains of *Discosia rubi* from eastern Texas showed anticryptococcal bioactivity with preferential activity in agar zone of inhibition assays against *C. neoformans* at 37°C versus 25°C. Assay-guided fractionation led to the purification and identification of chaetoglobosin P as the active component of these extracts. Genome sequencing of these strains revealed a biosynthetic gene cluster consistent with chaetoglobosin biosynthesis and β-methylation of the tryptophan residue. Proximity of genes of the actin-binding protein twinfilin-1 to the chaetoglobosin P and K gene clusters suggested a possible self-resistance mechanism involving twinfilin-1 which is consistent with the predicted mechanism of action involving interference with the polymerization of the capping process of filamentous actin. A *C. neoformans* mutant lacking twinfilin-1 was hypersensitive to chaetoglobosin P. Chaetoglobosins also potentiated the effects of amphotericin B and caspofungin on *C. neoformans*.

## Introduction

*Cryptococcus* species are among the most common causes of invasive fungal infections globally. There are an estimated 220,000 cases cryptococcosis annually and with mortality rates for patients with cryptococcal meningitis ranging from 10 to 70% (Rajasingham et al., 2017; Benedict et al., 2018). *Cryptococcus neoformans* and *Cryptococcus gattii* most often cause disease in people with compromised immune function. As many as a third of all HIV/AIDS-associated deaths are due to cryptococcal disease, exceeding the number due to tuberculosis. Current treatments are limited to few antifungal agents (amphotericin B, flucytosine, fluconazole), with no new therapies introduced in the past few decades. These options remain unsatisfactory because of their toxicity, inability to reliably eradicate the fungal pathogen, and the emergence of drug resistance (Brown et al., 2012; Perfect, 2017). Even when treated, cryptococcosis is further complicated by high recurrence rates and the need for long-term suppressive therapy. Furthermore, the few antifungal agents currently in clinical development have not been chosen based on effectiveness against *Cryptococcus* (Perfect, 2017; Van Daele et al., 2019). We hypothesize that a *Cryptococcus*-centric screening approach can result in identification of metabolites not generally detected in the well-worn *Candida albicans*-centric antifungal discovery paradigm (Xu et al., 2018). Therefore, new agents displaying anti-cryptococcal activity with minimal mammalian toxicity would be high priority objectives.

Chaetoglobosins are members of the cytochalasin family of fungal metabolites (Turner and Aldridge, 1983; Scherlach et al., 2010). The characteristic feature of cytochalasins is a substituted isoindole scaffold fused with a macrocyclic ring derived from a highly reduced polyketide backbone and an amino acid. Chaetoglobosins are biosynthesized from the amino acid tryptophan that is the source of the 10-(indol-3-yl) group fused to a non-aketide. Although biological effects of chaetoglobosins range widely, they are best known as microfilament-targeting molecules, and consequently they interfere with various cellular processes, including cytokinesis, intracellular motility, and exo- and endocytosis (Scherlach et al., 2010; Trendowski, 2014; Chen et al., 2020). Consequently, chaetoglobosins have been reported to have antifungal effects against a broad range of medically and environmentally relevant fungi (Zhao et al., 2017; Chen et al., 2020).

**FIGURE 1:**
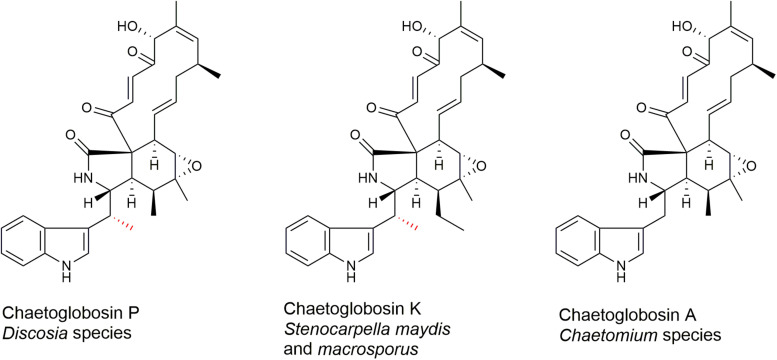
Molecular structures of chaetoglobosins P, K, and A. The methyl substitution on C-10 of chaetoglobosins P and K is indicated in red.

Chaetoglobosin A and chaetoglobosin variants have been described from *Chaetomium globosum* (Turner and Aldridge, 1983; Jiao et al., 2004; Xue et al., 2012; Chen et al., 2016; Jiang et al., 2016), other *Chaetomium* species (Sekita et al., 1982; Turner and Aldridge, 1983; Kawahara et al., 2013; Guo et al., 2019), *Cylindrocladium floridanum* (Ichihara et al., 1996), *Ijuhya vitellina* (Ashrafi et al., 2017), *Penicillium chrysogenum* (Zhu et al., 2017), *Penicillium expansum* (Andersen et al., 2004), *Phomopsis leptostromiformis* (Burlot et al., 2003), and *Stenocarpella macrospora* and *Stenocarpella maydis* (Cutler et al., 1980; Springer et al., 1980; Rogers et al., 2014). However, to the best of our knowledge, chaetoglobosin P has not been reported elsewhere since the original report (Donoso et al., 1997). Chaetoglobosin P (Figure 1) was characterized as a member of the chaetoglobosin family that differs from chaetoglobosin A (Figure 1) by a methyl substitution at the C-10 of the tryptophan residue (Donoso et al., 1997). The report described the producing strain TCF 9535 as a *Discosia* sp. and a method for purifying the compound from mycelial growth in a stirred fermenter. No details were given regarding how the strain was identified, its origin, or where it was deposited in a culture collection. No biological activity data on the compound was mentioned. The methyl-substituted tryptophan residue on C-10 is also found in chaetoglobosin K (Figure 1) from *S. macrospora* (Springer et al., 1980) and *S. maydis* (Wicklow et al., 2011), and in chaetoglobosins M and N from *P. leptostromiformis* (Burlot et al., 2003). A polyketide synthase-non-ribosomal peptide synthetase (PKS-NRPS) gene cluster, potentially responsible for encoding chaetoglobosin K, was identified in a draft genome sequence of *S. maydis* (Zaccaron et al., 2017).

Herein, we provide details on the producing strains of *D. rubi* (Ascomycota, Xylariales, Sporocadaceae) and their fermentation. We describe the bioactivity-guided purification and structure elucidation of chaetoglobosin P. Genome sequencing of the two producing strains has provisionally identified the chaetoglobosin P biosynthetic gene cluster (BCG) and revealed a possible mechanism of β-methylation of the tryptophan residue, thus leading to a biosynthetic hypothesis for chaetoglobosin P. Furthermore, we explored the antifungal spectrum of chaetoglobosin P in the fungal human pathogen *C. neoformans*, its potential mechanisms of action, and its synergy with other antifungal drugs.

## Materials and Methods

### Isolation and Characterization of *Discosia* Strains

Strain TTI-0863 was isolated by transferring mycelial fragments emerging from the cut end of a dead hardwood log onto cornmeal agar (Becton Dickinson) amended with streptomycin sulfate and chlortetracycline at 50 μg/mL. Strain TTI-0885 was recovered from mixed pine-hardwood forest litter using the dilution-to-extinction method (Torres et al., 2011). For morphological analysis and photography, strains were grown on oatmeal-wheat germ agar, V8 juice agar, and double-strength malt-yeast extract agar. Strains are available from the National Center for Agricultural Utilization Research Culture Collection (NRRL) as strains NRRL 66951 (TTI-0863) and NRRL 66952 (TTI-0885).

### General Experimental Procedures

Nuclear magnetic resonance (NMR) data were collected on a Bruker 500 MHz NMR instrument equipped with a 5-mm triple resonance cryoprobe at 298 K, with CDCl3 as solvent and tetramethylsilane as internal standard (δH/δC = 0). Semipreparative high performance liquid chromatography (HPLC) separations and HPLC-MS (mass spectrometry) data were acquired on an Agilent 1260 system equipped with a diode array detector (DAD) and coupled to an Agilent 6120 single quadrupole mass spectrometer, with a binary solvent system consisting of 0.1% aqueous formic acid (solvent A) and 0.1% formic acid in acetonitrile (solvent B).

### Growth Conditions

To produce mycelial extracts for agar zone of inhibition (ZOI) assays, strains TTI-0863 and TTI-0885 were grown on five media in 12-mL fermentations. For the seed cultures, cryopreserved agar plugs were grown for 7 days in YM agar plates (malt extract 10 g, yeast extract 2 g, agar 20 g, 1000 mL deionized H_2_O). Six agar discs from each culture were inoculated into 50 mL of SMY (maltose 40 g, neopeptone 10 g, yeast extract 10 g, agar 3 g, 1000 mL deionized H_2_O) in a 250-mL baffled flask. Seed cultures were grown at 24°C, 220 rpm for 4 days. One mL aliquots of the seed cultures were transferred to 50-mL EPA glass vials containing 12 mL of the following media: Wheat 1 medium (5.0 g whole wheat seeds, 8.5 mL of base liquid consisting of yeast extract 2.0 g, sodium tartrate 10.0 g, KH_2_PO_4_ 1.0 g, MgSO_4_.7H_2_O 1.0 g FeSO_4_.7H_2_O 0.050 g in 1000 mL deionized H_2_O); CYS80 (sucrose 80.0 g, yellow cornmeal 50.0 g, yeast extract 1.0 g in 1000 mL deionized H_2_O); MMK2 [mannitol 40.0 g, yeast extract 5.0 g, Murashige and Skoog Salts (Sigma-Aldrich M-5524) 4.3 g, in 1000 mL deionized H_2_O]; MPP [maltose 25.0 g, glucose 10.0 g, dried baker’s yeast 5.0 g, Pharmamedia (Archer Daniels Midland) 10.0 g, in 1000 mL deionized H_2_O], and MR (glucose 10.0 g, glycerol 10.0 g, sucrose 10.0 g, oat flour 5.0 g, soybean meal 20.0 g, primary yeast 10.0 g, CaCO_3_ 1.0 g, in 1000 mL deionized H_2_O). For TTI-0885, MPP medium was substituted for Supermalt (malt extract 50.0 g, yeast extract 10.0 g, FeSO_4_.7H_2_O 0.02 g, ZnSO_4_.7H_2_O 0.007 g, in 1000 mL deionized H_2_O), and MR for GGP media (10.0 glucose, 30.0 g glycerol, 5.0 Bacto peptone, 2.0 g NaCl in 1000 mL deionized H_2_O). Cultures in the Wheat 1 and CYS80 media were incubated statically with vials slanted at a 45° angle; the other media were agitated at 220 rpm. Fermentations were carried out at 24°C for 14 days, after which for each fermentation, 17 mL of 2-butanone was added and agitated at 220 rpm for 2 h. Eight mL of the organic phase was transferred to a glass vial and evaporated to dryness. The extracts were dissolved in 0.5 mL of dimethyl sulfoxide (DMSO) prior to assay. For a scaled-up cultivation, 100 mL of SMY seed media in a 500 mL flask was grown for 4 days at 23°C and 180 rpm, then 2.5 mL was transferred to each of ten 250-mL baffled flask containing 50 mL of MMK2 media. Fermentations were carried out at 24°C for 10 days.

### Two-Plate Thermal Sensitivity and Zone of Inhibition Assays

The assay for temperature-selective growth inhibition of *C. neoformans* was described previously (Xu et al., 2018). Overnight cultures of *Staphylococcus aureus* ATCC 43300 grown in Luria-Bertani (LB) broth (Sigma-Aldrich), and *C. albicans* ATCC 10231 and *C. neoformans* H99 grown in YM broth (1% malt extract, 0.2% yeast extract in deionized H_2_O) at 37°C were diluted with ATCC 43300 sterile H_2_O to an OD_600_ of 0.4 for *S. aureus* and 0.8 for *C. albicans* and *C. neoformans*. One-mL aliquots of the resulting mixture were combined with 35-mL aliquots of YMA (or LB agar for *S. aureus*) at 45°C and dispensed into one-well plates (Nunc Omnitray). Test wells (4 mm diam) were aspirated from the solidified plates with a syringe tip, and 10 μL of each extract in DMSO was applied to each well. Sets of fungal fermentation extracts were tested at a density of 20 extracts/plate in paired plates. One plate of each pair was incubated at 25 and 37°C. ZOIs were photographed after 48 h.

## Bioassay-Guided Microfractionation

The fungal extract from TTI-0863 grown in MMK2 medium was dissolved in DMSO (50 μL) and was diluted with an equal amount of methanol, filtered through 0.22 hydrophilic PTFE and analyzed by HPLC using an Agilent 1200 HPLC fitted with a 150 × 4.6 mm, 5 μm Ace Equivalence C18 column maintained at 35°C. The mobile phase consisted of (A) 0.1% formic acid in acetonitrile and (B) 0.1% aqueous formic acid. A gradient elution was used, from 10 to 100% A over 20 min, holding 100% for 4 min, 1.0 mL/min. The eluate was monitored by diode array detection (DAD) detection at 210, 254, and 310 nm, and 250-μL fractions collected directly in a 96-well plate using an automatic fraction collector. The contents of the 96-well plates were dried using a Genevac EZ-2 Evaporation System. A 3-μL aliquot of DMSO was transferred to each dried well, and the plate was gently agitated on a DPC Micromix 5 plate shaker. An overnight culture or *C. neoformans* H99 was diluted to an OD_600_ of 0.8 using sterile water. A 20-μL aliquot was combined with 10 mL of YM broth and 147 μL transferred to each well. The culture mixtures were gently homogenized, and incubated for 48 h at 37°C. After incubation, 15 μL of PrestoBlue Cell Viability Reagent (Invitrogen) was added and incubated for 16 h to visualize wells with growth inhibition.

### Isolation and Purification of Chaetoglobosin P

The mycelia and media were extracted by adding 50 mL of 2-butanone per flask followed by agitation at 200 rpm for 2 h. The extracts were combined, and mycelia separated by centrifugation (4000 rpm, 5 min, 20°C). The liquid phase was filtered, the organic phase separated and dried, yielding 0.89 g of extract. The extract was adsorbed in C_18_ resin and submitted to flash chromatography using a Grace Reveleris X2 flash chromatography system with a Reveleris C_18_ RP 40 g cartridge (50–100% MeOH over 12 min, flow rate 40 mL/min). Fractions containing the active compound were monitored by DAD at 280 nm, pooled and dried. Fractions were further purified by semi-preparative HPLC using an Agilent Zorbax SB-C_18_ column; 5 μm; 9.4 × 250 mm; with a gradient of 40-85% A for 11.2 min (solvent A, 0.1% formic acid in acetonitrile, A, solvent B, 0.1% formic acid in H_2_O; 4.0 mL/min). Preparative HPLC yielded 12.1 mg of chaetoglobosin P.

### Minimal Inhibitory Concentration and Checkerboard Assays

To quantify the inhibitory concentrations of chaetoglobosins for strains of fungal pathogens, minimal inhibitory concentrations (MICs) were measured using species-specific modifications to Clinical and Laboratory Standards Institute (CLSI) standard methods (Alexander, 2017). For each microorganism, cells or spores were sequentially diluted in phosphate-buffered saline (PBS) and RPMI-1640 medium buffered with MOPS (Sigma-Aldrich) to a final concentration of 500 cells/mL or 20,000 spores/mL. The final suspension was incubated with serial dilutions of drug at a dose range of 0.049–50 μg/mL. Cells were grown for 2 days (*C. albicans* and *Aspergillus fumigatus*) or 3 days (*C. neoformans*) at 37°C.

To measure viability, the indicator dye Alamar Blue (Bio-Rad) was added 24 h prior to fluorescence detection (BMG FLUOStar Optima plate reader). MIC_50_ values based on Alamar Blue absorbance were determined as described by the manufacture and by comparing values treated versus untreated control samples. Additionally, growth dynamics and cellular morphogenesis was assessed microscopically every 24 h for all experimental samples. Antifungal drug synergy was assessed by standard checkerboard MIC testing as previously described, using the fractional inhibitory concentration (FIC) index to assess for drug synergy (Pianalto et al., 2019) but modified by the sample preparation and Alamar Blue measurement as described for MIC determination. In addition, DMSO drug solvent concentration was maintained at 1% for all samples and experiments to avoid DMSO-induced artifacts.

### Visualization of Actin Binding

Wild type strain *C. neoformans* H99 was incubated overnight in YPD medium (yeast extract 20 g, peptone 10 g, glucose 20 g, 1000 ml deionized H_2_O) at 30°C, diluted to OD 0.2 in YPD, incubated 2 h at 30°C, then incubated for 4 h at 37°C with the indicated drug or solvent. After treatment, cells were fixed by adding 1/4 volume of formaldehyde for 30 min, permeabilized with 1% Triton X-100 in PBS, then incubated 3 h with tetramethylrhodamine (TRITC)-conjugated phalloidin (0.5 μg/mL final concentration; Sigma-Aldrich P-1951) which binds to F-actin structures. Fluorescent and DIC microscopy images were captured at 680 × magnification using a Zeiss Imager A1 microscope equipped with a Zeiss Axio-Cam MRM digital camera.

### *Cryptococcus neoformans* Library Screen

To screen for mutants that were either resistant or hyper-sensitive to chaetoglobosin P, a library of 3880 *C. neoformans* loss-of-function mutant strains (Liu et al., 2008) was replicate-plated and incubated in YPD medium in 96-well plates. Strains were diluted 200-fold in PBS then 20-fold into YNB medium (BD-Difco) containing the viability indicator resazurin blue (0.002%) (Monteiro et al., 2012). The strains were incubated with chaetoglobosin P at either 25 μg/ml (resistance screen) or 1.56 μg/mL (super-sensitive). 96-well plates were incubated at 37°C (resistant) or 30°C (supersensitive). Each test plate included a no-drug plate incubated at 30°C and 37°C. In YNB medium, resazurin blue quickly reduced from fluorescent resorufin to dihydroresorufin, a colorless product. Lack of blue or pink color coupled with growth compared to wild type determined resistant and hypersensitive mutants. As a control, the library was incubated in parallel in the absence of drug to ensure vigorous growth of all strains.

### Genome Sequencing and Annotation of Putative Chaetoglobosin Gene Clusters

Strains TTI-0863 and TTI-0885 were grown in a static culture of 100 mL SMY for 14 days at 23°C.

Mycelium was filtered, pressed dry, frozen at −80°C, and lyophilized. Genomic DNA was purified from ground mycelial powder with a Zymo Research Corporation Quick-DNA^TM^ Fungal/Bacterial Miniprep Kit. For preparation of sequencing libraries, 500 ng of total genomic DNA were used as the template and processed using the KAPA HyperPlus Kit for PCR-free workflows (Roche, Switzerland) according to the manufacturer’s instructions. Sequencing libraries were size selected for 600–800 bp fragments using a LightBench (Coastal Genomics, Canada). Whole genome sequencing was run on a HiSeq 4000 Sequencing System (IL, United States). The genome was assembled by SPAdes using standard parameters (Bankevich et al., 2012).

The chaetoglobosin gene clusters in TTI-0863, TTI-0885 (GenBank MT459796), *S. maydis* (Bioproject PRJNA397884) and *C. globosum* (Bioproject PRJNA12795) were predicted by submitting the unannotated scaffold sequences for antiSMASH analysis^1^ (Blin et al., 2019) and by reciprocal BLAST searches of predicted proteins and annotated scaffolds with sequences from previously determined core genes of the chaetoglobosin gene clusters from *C. globosum* (GenBank Bioproject PRJNA16821) and *S. maydis* (Zaccaron et al., 2017). The ORFs of the BGCs were refined by a combination of gene predictions from Augustus (Stanke et al., 2006) and FGENESH (Solovyev et al., 2006) using *Magnaporthe grisea* as the reference genome followed by manual correction of ORFs. Orthologous BCGs from TTI-0865, TTI-0885, *S. maydis* and *C. globosum* were aligned using Easyfig (Sullivan et al., 2011) and plotted to illustrate gene identity between orthologs (%) and their comparative microsynteny.

### Phylogenetic Tree Construction

To reconstruct the approximate phylogenetic position of strain TTI-0863 and TTI-0885, genomic DNA was purified from lyophilized mycelial powder with a Zymo Research Corporation Quick-DNA^TM^ Fungal/Bacterial Miniprep Kit. The rDNA region containing the internal transcribed spacer (ITS) region and the partial sequence of 28S rDNA was amplified with primers ITS1 and LR7. Purified PCR products were cloned in the pJET1.2/blunt cloning vector (Thermo Fisher Scientific, MA, United States) and sequenced using primer LR0R and pJET1.2 forward and reverse sequencing primers, supplied by CloneJET PCR cloning kit (Thermo Fisher Scientific) from GENEWIZ, Inc. (South Plainfield, NJ, United States). Partial sequences obtained from sequencing reactions were assembled with Geneious (version R11^2^).

The phylogenetic tree was constructed based on alignments of the ITS region (Genbank MT452903, MT452904). The DNA sequences from other *Discosia* species (Tanaka et al., 2011; Liu F. et al., 2019) were aligned by using ClustalW implemented in MEGA 7.0 (Kumar et al., 2016), and the resulting alignment was manually trimmed. Phylogenies were inferred by the maximum likelihood (ML) method implemented in MEGA 7.0 under a K2 + G model. Bootstrap supports were calculated using the default options in MEGA 7.0 with 1000 replicates per run. To construct the twinfilin-1 tree, putative twinfilin-1 proteins encoded by TTI-0863 and TTI-0885 and twinfilin-1 from *C. globosum* were used as queries in BLAST searches against the NCBI databases. Protein sequences were aligned by using ClustalW implemented in MEGA 7.0, and the resulting alignment was manually adjusted. Phylogeny of protein sequences was inferred by the ML method implemented in MEGA 7.0 under a LG + G model. Bootstrap supports were calculated using the default options in MEGA 7.0 with 1000 replicates per run.

## Results and Discussion

### Identification and Characterization of Chaetoglobosin P-Producing Strains

Two strains (TTI-0863 = NRRL 66951, TTI-0885 = NRRL 66952) of an unknown *Discosia* species were isolated from mycelium emerging from the cut end of a dead hardwood log and from mixed pine and oak forest litter at the Center for Biological Studies Field Studies, Sam Houston State University, near Huntsville, Walker Co., Texas. Cultures on malt-yeast extract and oatmeal-wheatgerm agar grew as light to dark gray to olive-gray wooly colonies (Supplementary Figures S1A–C). The mycelia eventually formed patches of black stromatic tissue after one to two weeks (Supplementary Figure S1A–E). Black sporodochial conidiomata formed on some of these stromata after 4–5 weeks. The internal surfaces of the conidiomata were lined with a dense layer of cylindrical conidiogenous cells. The conidia arise by holoblastic conidiogenesis, and conidia succeed from the conidiogenous locus by fission of a double septum. The conidia were thin-walled, colorless, 15–21 × 2–5 μm, mostly 3-septate, with a truncate basal scar, and with two or one subapical filiform setulae up 12 μm long arising from the concave side of the conidia (Supplementary Figures S1E,H). Their morphological features were typical of fungi of the genus *Discosia* (Sutton, 1980; Nag Raj, 1993). BLAST searches with rDNA sequences from the ribosomal ITS region and large subunit gene retrieved many *Discosia* sequences with a similarity of >98%. A ML tree based on an alignment of ITS sequences (Supplementary Figure S2) placed strains TTI-0863 and TTI-0885 in clade (81% bootstrap support) with the type strains of *Hyphodiscosia radicicola* (Watanabe, 1992) and *D. rubi* (Liu F. et al., 2019). The identity of the ITS region of these strains with the type strain of *D. rubi* was 99.8% (100% coverage). The sequences of the RNA polymerase II second largest subunit (*rpb2*), the translation elongation factor 1-α (*tef-1*α), and β-tubulin (*tub2*) genes from the type strain of *D. rubi* showed 100, 100, and 99.3% identity, respectively, to the corresponding genes from genome sequences of TTI-0863 and TTI-0885. Furthermore, cultural characteristics, conidiogenesis, and conidial morphology were very similar to that described for *D. rubi* (Liu F. et al., 2019; Supplementary Figures S1F–H). Thus, we concluded that TTI-0863 and TTI-0885 were conspecific with *D. rubi*. However, we should qualify this identification by noting that nearly 100 species have been described in the genus *Discosia* (Sutton, 1980; Nag Raj, 1993). Only a small proportion of them have been cultured and undergone modern morphological and phylogenetic analysis, so the name these strains and possibly as well as *D. rubi* eventually may be found to be the same as one of the previously unstudied species.

### Detection and Isolation of Chaetoglobosin P From Fermentations of Strains TTI-0863 and TTI-0885

Natural products extracts from fungi were systematically tested for temperature selective activity in a two-plate agar to identify agents with enhanced of growth inhibition at ≥37°C, an effect that could potentially mitigate *in vivo* virulence of *C. neoformans* (Xu et al., 2018). Temperature-dependent *C. neoformans* bioactivity was observed from the extracts of TTI-0863 and TTI-0885 when grown in wheat and MMK2 (also in medium supermalt for TTI-0885) where bioactivity was enhanced at 37°C compared to the same assay at 25°C (Supplementary Figure S3). Bioactivity-guided microfractionation of the TTI-0863 MMK2 extract showed the presence of a major active compound. LC-MS analysis allowed the characterization of the active peak as a pure compound with MW = 542 g/mol and λ_max_ 226 and 278 nm. Chromatographic separations from a 500-mL batch culture of TTI-0863 in medium MMK2 afforded a yellow solid from which a complete set of NMR data was obtained (Supplementary Figures S4–S8). Analysis of obtained spectra closely matched exactly previous data reported for chaetoglobosin P (Table 1; Donoso et al., 1997).

**TABLE 1:** Comparison of 1H and 13C NMR chemical shifts of chaetoglobosin P purified in this study and the original data reported by Donoso et al. (1997).

Number	Chaetoglobosin P (Donoso et al., 1997)		Chaetoglobosin P (this study)	
	^13^C	^1^H	^13^C	^1^H
1	173.3		173.2	
2		5.67 (1H, br s)		5.61 (1H, s)
3	56.4	3.81 (1H, m)	56.4	3.83 (1H, m)
4	45.4	3.06 (1H, m)	45.4	3.08 (1H, m)
5	36.4	1.82 (1H, br q, J = 7.1 Hz)	36.4	1.85 (1H, dq, J = 5.5, 7.1 Hz)
6	57.8		57.9	
7	62.3	2.76 (1H, d, J = 5.2 Hz)	62.2	2.78 (1H, d, J = 5.3 Hz)
8	49.5	2.76 (1H, dd, J = 5.2, 10 Hz)	49.5	2.14 (1H, dd, J = 5.2, 9.9 Hz)
9	63.7		63.7	
10	36.4	3.06 (1H, m)	36.4	3.09 (1H, m)
11	13.7	1.25 (3H, d, J = 7.1 Hz)	13.7	1.15 (3H, d, J = 7.1 Hz)
12	19.8	1.25 (3H, s)	19.8	1.26 (3H, s)
13	128.3	6.05 (1H, dd, J = 10.2, 11.4 Hz)	128.5	6.08 (1H, ddd, J = 1.1, 10.6, 15.0 Hz)
14	133.5	5.21 (1H, ddd, J = 3.8, 10.2, 14.7 Hz)	133.7	5.22 (1H, ddd, J = 4.1, 9.9, 15.2 Hz)
15	42	2.24 (1H, m), 2.04 (1H, m)	42.0	2.27 (1H, m), 2.03 (1H, m)
16	32	2.40 (1H, m)	32.1	2.43 (1H, m)
17	140.4	5.60 (1H, d, J = 8.6 Hz)	140.6	5.62 (1H, dd, J = 1.2, 10.9)
18	132.4		132.5	
19	81.7	5.00 (1H, br s)	81.8	5.03 (1H, d, J = 4.4 Hz)
20	201.8		201.8	
21	131.1	6.49 (1H, d, J = 16.7 Hz)	131.2	6.5 (1H, d, 16.7 Hz)
22	136.6	7.67 (1H, d, J = 16.7 Hz)	136.5	7.69 (1H, d, J = 16.7 Hz)
23	197.6		197.7	
1’		8.09 (1H, br s)		8.11 (1H, s)
2’	121.8	6.92 (1H, d, J = 2.2 Hz)	121.9	6.94 (1H, d, J = 2.3 Hz)
3’	116.8		116.9	
3’a	126		126.0	
4’	118.7	7.50 (1H, d, J = 7.5 Hz)	118.8	7.52 (1H, d, J = 7.7 Hz)
5’	122.6	7.12-7.16 (1H, m)	122.7	7.17 (1H, ddd, J = 0.9, 7.5, 7.7 Hz)
6’	120	7.12-7.16 (1H, m)	120.1	7.15 (1H, ddd, J = 0.9, 7.5, 7.8 Hz)
7’	111.6	7.31 (1H, d, J = 7.3 Hz)	111.8	7.33 (1H, d, J = 7.8 Hz)
7’a	136.6		136.7	
10’	13.7	1.13 (3H, d, J = 7.0 Hz)	13.7	1.27 (3H, d, J = 7.1 Hz)
16’	21	0.99 (3H, d, J = 6.9 Hz)	21.1	1.01 (3H, d, 6.8 Hz)
18’	10.6	1.30 (3H, s)	10.6	1.32 (3H, d, 1.0 Hz)
OH-19		3.81 (1H, m)		3.82 (1H, s)

### Antifungal Activity of Chaetoglobosin P

The MIC of chaetoglobosin P for *C. neoformans* H99 at 37°C was 6.3 μg/mL and 69.5 μg/mL at 25°C. The compound demonstrated less potent antifungal activity against other fungal pathogens (*A. fumigatus* 12.5 μg/mL and *C. Albicans* >50 μg/mL) and did not affect growth of *S. aureus* at the highest concentration tested (128 μg/mL, 69.5 μM).

**FIGURE 2:**
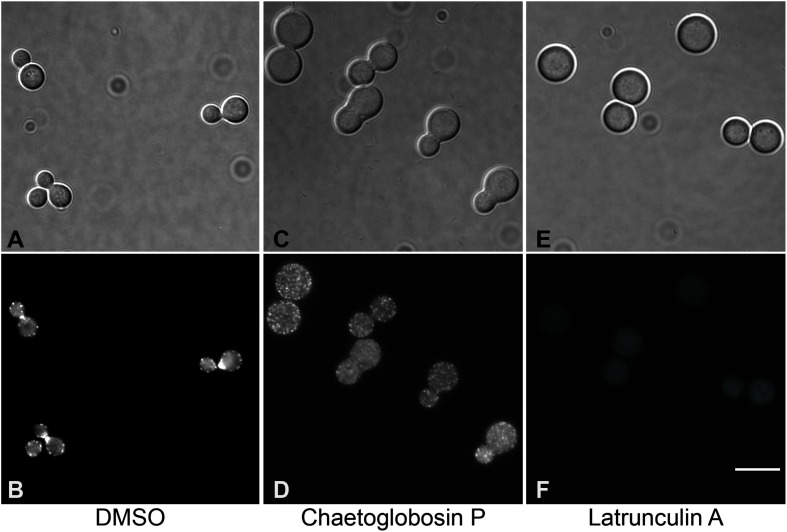
Visualization of actin polymerization and effects of inhibitors in cells of *C. neoformans*. Cells were incubated overnight in YPD medium at 30°C, diluted to OD 0.2 in YPD, incubated 2 h at 30°C, then incubated for 4 h at 37°C with the indicated drug or 1% DMSO. After treatment, cells were formaldehyde fixed, permeabilized, then incubated with TRITC-conjugated phalloidin which binds to F-actin structures. **(A,B)** DMSO, negative control. **(C,D)** Treatment with chaetoglobosin P. **(E,F)**. Latrunculin A, positive control.

To further assess the antifungal mechanism of action for chaetoglobosin P, we first explored its previously reported activity to inhibit actin microfilament formation. Wild-type *C. neoformans* cells were incubated in a rich medium at 30°C to mid-logarithmic phase. They were then treated with either chaetoglobosin P (25 μg/mL), the actin inhibitor latrunculin A (100 μM), or DMSO solvent alone (1%). The strains were incubated at 37°C for an additional 4 h, formalin fixed, permeabilized, and incubated with TRITC-conjugated phalloidin to visualize filamentous actin (F-actin) localization. The cells treated with DMSO alone demonstrated a typical pattern of actin polarization associated with budding and cell division. In these cells, F-actin was localized primarily at the site of cell division/separation, and as puncta at the distal ends of the budding cells. In contrast, the cells treated with chaetoglobosin P demonstrated a non-polarized pattern of actin localization in cortical punctate patches (Figure 2). This result was similar to prior observations in *C. neoformans* of failed actin polarization in the absence of Ras1 or Cdc42 activity (Nichols et al., 2007; Ballou et al., 2010). A similar pattern of actin mislocalization can also be seen when *Saccharomyces cerevisiae* is treated with occidiofungin, an actin-binding bacterial glycopeptide (Ravichandran et al., 2019). Treatment of *C. neoformans* with the actin inhibitor latrunculin A, which inhibits polymerization of actin monomers into F-actin, resulted in a distinct pattern of staining in which no organized foci of filamentous actin were observed (Figure 2).

**FIGURE 3:**
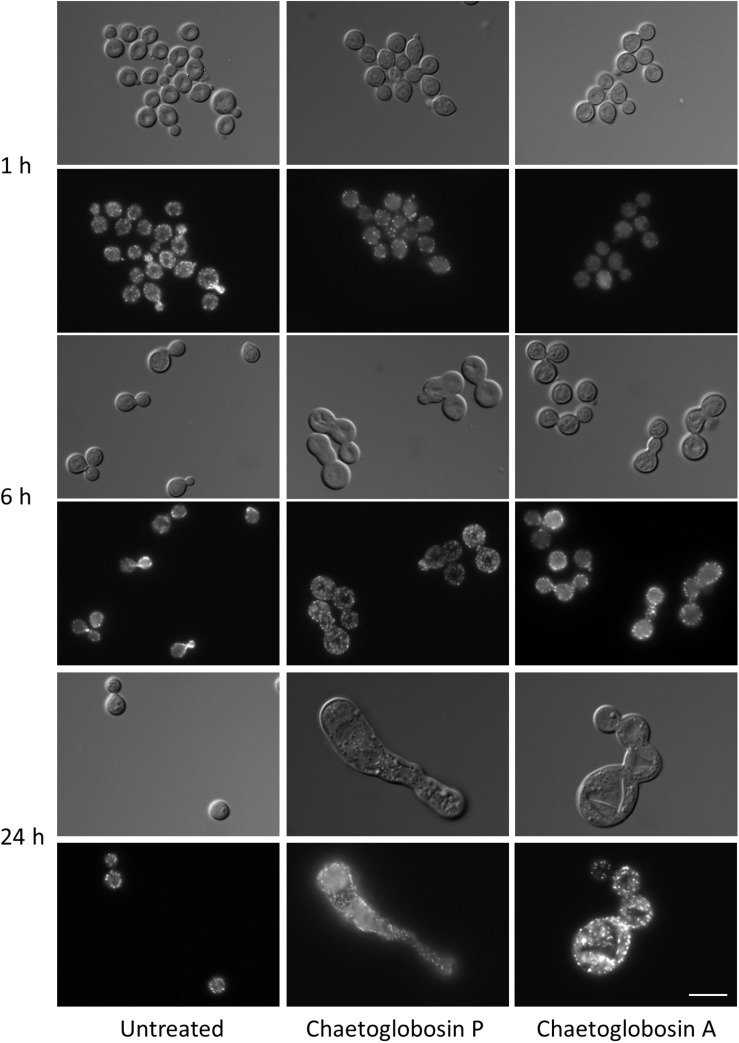
Effects of chaetoglobosins P and A on morphology and actin localization in cells of *C. neoformans*. Wild-type strain H99 was incubated overnight in YPD at 30°C, diluted to OD 0.2 in YPD medium, incubated 2 h at 30°C, then incubated at 37°C with the indicated drug. At the indicated time, cells were formaldehyde-fixed, permeabilized, and incubated with TRITC-conjugated phalloidin which binds to actin structures. Bar = 10 μm.

We also assessed actin localization and resulting cellular effects of chaetoglobosins P and A over a time course (Figure 3). Untreated *C. neoformans* cells demonstrated expected patterns of phalloidin staining during the cell budding cycle, consistent with actin’s role directing dynamic cytoskeletal reorganization. F-actin was primarily localized in cortical puncta during isotropic growth, polarizing to the sites of newly forming buds and cell separation. Actin polarization and cable formation was absent in cells treated with either chaetoglobosin P or chaetoglobosin A, even after one hour of compound exposure (Figure 3). F-actin localization was limited to unpolarized surface puncta throughout the remainder of the 24-h period of observation. During this time, the cells exhibited increasing defects in morphogenesis, including, swelling, growth as elongated cells or as chains due to failed cell separation (Figure 3).

We screened a library of 3880 *C. neoformans* loss-of-function mutant strains (Liu et al., 2008) for altered drug resistance compared to wild-type (Supplementary Table S1). Of 294 chaetoglobosin P-resistant strains (Supplementary Tables S1, S2), 135 had mutations in genes of unknown function. Among the resistant strains were several groups of mutants that function in similar cellular process and signaling pathways. For example, resistant strains (Supplementary Table S1) included those with mutations in the *TCO1*, *TCO5*, *TCO6*, and *TCO7* genes encoding “two-component-like” signaling elements that function upstream of the *C. neoformans* Hog1 stress response pathway (Bahn et al., 2006). Interestingly, *C. neoformans tco* mutants are also relatively resistant to fludioxonil, an antifungal compound that mediates microbial morphogenesis by inhibiting transport-associated phosphorylation of glucose (Bahn et al., 2006). Other resistant strains included those with mutations in cAMP/PKA signaling proteins (Gpa1, Cac1, Pka1, and Crg1); those with mutations in protein secretion/trafficking (Sec104-1, Sec104-2, Vps17, Vps28, kinesin, dynein, and the vesicle transporter Sft2B); and strains with predicted defects in Ras/morphogenesis pathways required for efficient actin polarization in response to stress (Ras2, Rho104, Rho-GEF, Ran, Rab, and the regulator of G-protein signaling Rgs1). The large number of resistant mutants, with defects in several different cell processes and signaling pathways, suggests that there is a low barrier to resistance for this compound at the tested screening concentration.

Additionally, 26 mutant strains demonstrated marked hyper-susceptibility to this compound (Supplementary Table S1). Of these, 15 had mutations in genes with varied predicted biological function, including the cell polarity protein Mor2, the actin-related chromatin remodeling protein Arp6, and the Stp1 two-site protease that activates the sterol homeostasis pathway. Additionally, 11 of these strains had mutations in genes encoding proteins of unknown function.

### Synergy of Chaetoglobosin P With Antifungal Drugs

To further explore the translational relevance of the antifungal activity of chaetoglobosin P, we tested the ability of this compound to offer additive or synergistic activity to existing antifungal compounds. Using broth dilution checkerboard assays, we calculated the FIC of the drugs in combination, defining drug synergy (FIC < 0.5), additivity (FIC 0.5–1), indifference (FIC 1–4), and antagonism (FIC >4). When used in combination against *C. neoformans*, chaetoglobosin P demonstrated antifungal synergy with amphotericin B (FIC = 0.45). Interestingly, a synergistic antifungal effect was also observed for chaetoglobosin P and amphotericin B for *C. albicans* and *A. fumigatus* (FIC 0.3 and 0.4, respectively). The chaetoglobosins also demonstrated synergy in *C. neoformans* with the cell wall-active antifungal caspofungin (FIC 0.3), with a subinhibitory concentration of chaetoglobosin A (1.5 μM) reducing the MIC of caspofungin fourfold (12.5 to 3.1 μM). This result is interesting given the relative tolerance of this fungus to the echinocandins (Thompson et al., 1999). No additive or antagonistic effect was observed with chaetoglobosin P co-treatment of *C. neoformans* with either fluconazole (FIC = 1) or itraconazole (FIC = 1).

### Identification of Putative Chaetoglobosin P Biosynthetic Gene Cluster

**FIGURE 4:**
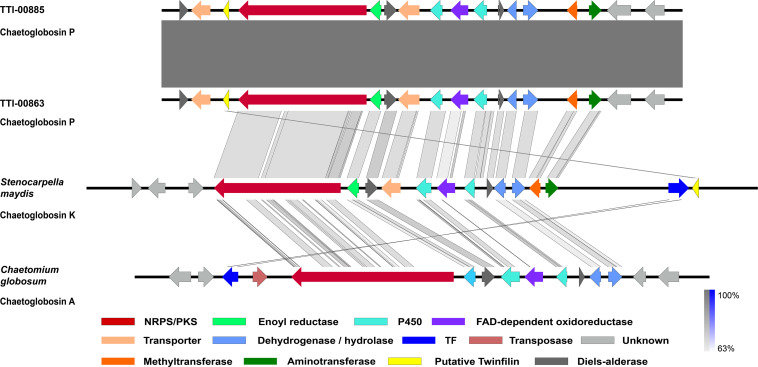
Graphic representation of the putative and proven chaetoglobosin-type gene clusters and their microsynteny. Chaetoglobosin P gene cluster from *Discosia rubi* strains, the chaetoglobosin K gene cluster from *Stenocarpella maydis* A1-1, and the chaetoglobosin A gene cluster from *Chaetomium globosum* CBS 148.51. The intensity of the gray scale bar indicates the degree of nucleotide similarity.

To understand how the biosynthesis of chaetoglobosin P differed from chaetoglobosin A, we prepared draft genome sequences of TTI-0863 and TTI-0885. A BLAST search of protein models from TTI-0863 and TTI-0885 with the chaetoglobosin A PKS-NRPS protein sequence from *C. globosum* CBS 148.51 (CHGG_01239 from PRJNA16821) retrieved a PKS-NRPS gene along with 13 colocalized genes partially resembling the gene order, content and predicted function of the chaetoglobosin A BGC (Table 2 and Figure 4). However, the gene content and gene order were more similar to the putative chaetoglobosin K gene cluster from *S. maydis* A1-1 with 12 of 13 orthologs localized the same order (PRJNA397884) (Zaccaron et al., 2017). Thus, triangulation between the chaetoglobosin A and putative chaetoglobosin K BGCs indicated with a high degree of confidence that these BGCs in TTI-0863 and TTI-0885 are responsible for encoding chaetoglobosin P biosynthesis.

**TABLE 2:** Predicted proteins from the putative chaetoglobosin P gene cluster in *Discosia rubi* TTI-0885 and their similarity to orthologs in the chaetoglobosin A gene cluster from *Chaetomium globosum* CBS 148.51.

Gene in *D. rubi*	Putative function	Gene name in *C. globosum*	Similarity%
g66	Twinfilin-1	Absent	–
g67-1	PKS-NRPS hybrid	CHGG_01239	46.8
g67-2	Enoyl reductase	CHGG_01240	61.2
g68	Diels-alderase	CHGG_01241	68.3
g69	MFS general substrate transporter	Absent	–
g70	FAD-dependent oxidoreductase	CHGG_01242-2	49.4
g71	P450	CHGG_01242-1	47.3
g72	P450	CHGG_01243	59.5
g73	Hypothetical protein	CHGG_01244	44.7
g74	Short chain dehydrogenase	CHGG_01245	59.7
g75	Alpha/beta hydrolase	CHGG_01246	68.3
g76	C-methyltransferase	Absent	–
g77	Branched chain amino-acid aminotransferase	Absent	–

Chaetoglobosins P and K are distinguished from chaetoglobosin A and its variants by β-methylation of the C-10 of the tryptophan residue (Figure 1). β-methyltryptophans [(2S,3S)-β-MeTrp, (2S,3R)-β-MeTrp] are important building blocks for actinomycete natural products. (2S,3S)-β-methyltryptophan is found in metabolites such as, maremycins, Fr99452, lavendamycin, and streptonigrin (Liu B. et al., 2019), and (2S,3R)-β-MeTrp is incorporated into indolmycin and telomycin. Formation of the (2S,3S)-β-methyltryptophan biosynthetic precursor of streptonigrin (Xu et al., 2013; Kong et al., 2016) is catalyzed by enzymes encoded by the *stnR-stnQ1-stnK3* cassette. Similarly, the *marG-marH-marI* gene cassette of the maremycin pathway from *Streptomyces* sp. B9173 encodes the enzymes that catalyze the stereospecific formation of (2S,3S)-β-methyltryptophan from L-tryptophan (Liu B. et al., 2019). The conserved biosynthetic cluster consists of a PLP-dependent aminotransferase (stnR/MarG), a cupin-fold protein (StnK3/MarH) and a SAM-dependent C-methyltransferase (StnQ1/MarI). MarH is responsible for the stereochemical epimerization of the methyl group. In contrast, (2S,3R)-β-methyltryptophan residues are formed by a two-enzyme pathway in indolmycin in *Streptomyces griseus* (Du et al., 2015) and telomycin in *Streptomyces canus* (Fu et al., 2015), and these enzymes show high homology to MarG and MarI, respectively. In fungi, β-methyl-tryptophan [(2S,3R)-β-MeTrp] has a limited distribution and is found in chaetoglobosins K, M, N, and P. Two predicted genes present in both the putative chaetoglobosin P and K BGCs, but absent in the chaetoglobosin A cluster, are those for an a C-methyltransferase and aminotransferase (Figure 4 and Table 2, G76, G77). A search of the Protein Database (PDB) with gene G76 recognized a 2-ketoarginine methyl transferase domain (ketoArg_3Met) characteristic of SAM-dependent C-methyltransferases. Searches with gene G77 recognized that it belongs to the pyridoxaL 5′-phosphate dependent enzymes class IV (PLPDE_IV) characteristic of consists of branched-chain amino acid aminotransferases, Plausibly, these genes could encode enzymes necessary for the formation of at β-methyltryptophan chaetoglobosin precursor.

**FIGURE 5:**
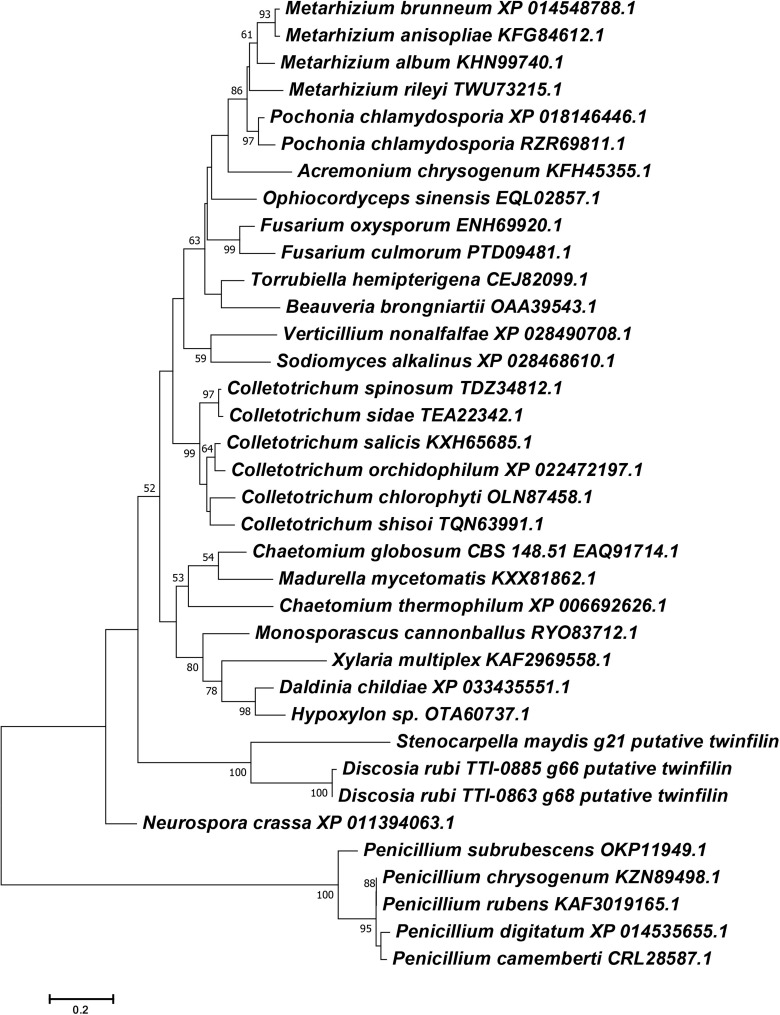
Phylogenetic reconstruction of selected Ascomycete twinfilin-1 protein sequences. Phylogeny was inferred by the ML method implemented in MEGA 7.0 under a LG + G model. Bootstrap supports >50% from 1000 replicates per run are labeled on branch nodes. *Penicillium* species were designated as the outgroup.

Another surprising feature shared by the putative chaetoglobosin P and K gene clusters but absent in the chaetoglobosin A gene cluster was the presence of a gene resembling eukaryotic twinfilin-1 (*twf1*) (Figure 4) either adjacent to the core PKS-NRPS gene within the gene cluster (chaetoglobosin P) or immediately upstream (chaetoglobosin K). In the draft genome of the chaetoglobosin A producing fungus, *C. globosum*, the *twf1* gene occurs on a different contig from the chaetoglobosin A gene cluster, and its predicted protein sequence mirrors the relative phylogenetic position of *C. globosum* among the Sordariomycetes (Figure 5). In both *D. rubi* and *S. maydis*, the BGC-associated copy of *twf1* was the only copy that could be detected in any of the three genomes. Thus, in these fungi, the *twf1* gene did not follow the canonical pattern where upregulation of a divergent BGC-associated resistance gene compensates for a separate primary housekeeping gene (Kjærbølling et al., 2019). Immediate proximity to a chaetoglobosin BGC would facilitate coregulation with metabolite production. This gene had high sequence similarity to orthologous fungal genes tentatively identified as *twf1* (Figure 5). Phylogenetic analysis of the chaetoglobosin-associated twinfilin-1 protein sequences from *D. rubi* and *S. maydis* showed that they formed a distinct and statistically significant clade (100% bootstrap value) outside their predicted positions among related Sordariomycete twinfilin-1 proteins suggesting that these proteins have evolved specific sequence variants as a consequence of for selection for chaetoglobosin biosynthesis (Figure 5).

Twinfilin proteins bind actin monomers, preventing their assembly into actin filaments. These proteins also participate in the protective capping of the distal ends of actin filaments (Johnston et al., 2018). It would, therefore, be predicted that *twf1* overexpression during chaetoglobosin biosynthesis might competitively block the binding of chaetoglobosins to the barbed, fast-growing ends of actin filaments. Similarly, we hypothesized that *twf1* deletion mutants would lack an intact actin capping complex and be hypersusceptible to the effects of chaetoglobosins. Consistent with this hypothesis, a *C. neoformans* strain with a loss of function mutation in the predicted *twf1* gene (CNAG_03864) displayed a strikingly reduced MIC at 37°C for chaetoglobosin P (<0.39 μg/mL, compared to 6.25 μg/mL for wild-type parent). Moreover, the *C. neoformans twf1*Δ mutant demonstrates an enhanced effect compared to wild-type in dose-dependent cell cycle defects observed after incubation with either chaetoglobosin P or chaetoglobosin A (Supplementary Figure S9). Together, these results support a model in which chaetoglobosin P binds the distal end of actin filaments, inhibiting their dynamic assembly/disassembly, and resulting in the arrest of actin-mediated cell division and morphogenesis (Supplementary Figure S9). We predict in future experiments that the overexpression of twinfilin-1 proteins encoded by the *twf1*-like genes in the chaetoglobosin gene cluster may offer the producing strains relative protection from the effects of this compound compared to other sensitive fungi or other eukaryotic organisms.

## Conclusion

We have provided new information on the origin, biology, biosynthesis, and antifungal effects of the poorly known fungal secondary metabolite, chaetoglobosin P. New producing strains were identified as *D. rubi* and made available at the NRRL culture collection. New data on antifungal activity, synergy with other antifungal drugs, morphological effects, and mode of action in *C. neoformans* are provided. Genome sequencing of the producing strains has provisionally identified a BGC for chaetoglobosin P that is highly similar to the putative gene cluster encoding chaetoglobosin K from the maize pathogen *S. macrospora*. These two BCGs share novel genes that could encode enzymes for synthesis of their unusual non-proteinogenic amino acid, β-methyltryptophan. Proximity of the *twf1* gene to these BCGs suggests a hypothetical mode of self-resistance to their own toxic metabolite.

## Data Availability Statement

The datasets presented in this study can be found in online repositories. The names of the repository/repositories and accession number(s) can be found in the article/Supplementary Material.

## Author Contributions

BP, CN, NL, PW, CH, JA, and GB contributed to acquisition, analysis, and interpretation of data for the work. All authors contributed to the design of the work, writing and revision of the final draft, and approved the submitted and final version.

## Conflict of Interest

PW and CH are employees of Hexagon Bio, and genome sequencing of strains was supported and carried out by Hexagon Bio. The remaining authors declare that the research was conducted in the absence of any commercial or financial relationships that could be construed as a potential conflict of interest.

## Footnotes

1 https://fungismash.secondarymetabolites.org/

2 https://www.geneious.com/
