# Supplementary material for: Identification of the Antifungal Metabolite Chaetoglobosin P From Discosia rubi Using a Cryptococcus neoformans Inhibition Assay: Insights Into Mode of Action and Biosynthesis
Source: Front Microbiol. 2020 Jul 28;11:1766. doi: 10.3389/fmicb.2020.01766 (PMC7399079; doi:10.3389/fmicb.2020.01766)
Supplement: Supplementary file 1 [file Data_Sheet_1.docx]

**Supplemental Materials**

**Frontiers in Microbiology**

**Identification of the antifungal metabolite chaetoglobosin P from *Discosia rubi* using a *Cryptococcus neoformans* differential temperature sensitivity assay**

***Bruno Perlatti^1^, Connie B. Nichols^2^, Lan Nan^1^, Philipp Weinmann^3^, Colin J.B. Harvey^3^, J. Andrew Alspaugh^2^, Gerald F. Bills^1,*^***

^1^ Texas Therapeutics Institute, The Brown Foundation Institute of Molecular Medicine, University of Texas Health Science Center at Houston, Houston, Texas 77054, USA

^2^ Departments of Medicine and Molecular Genetics and Microbiology, Duke University Medical Center, Durham, North Carolina 27710, USA

Hexagon Bio, Menlo Park, California 94205, USA

* Corresponding author: [gerald.f.bills@uth.tmc.edu](mailto:gerald.f.bills@uth.tmc.edu)


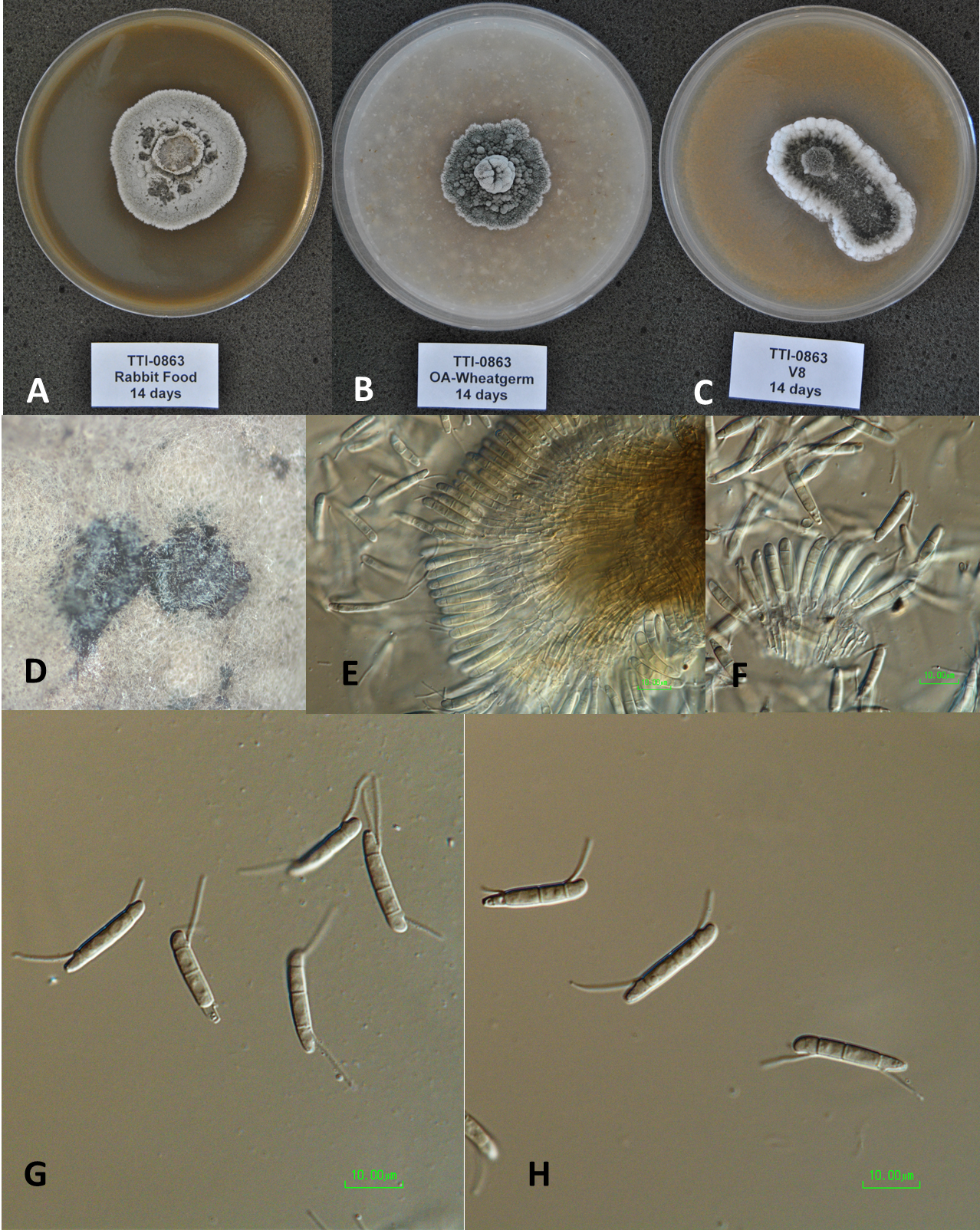
Figure S1. *Discosia rubi* TTI-0863. A. Culture on rabbit food agar. B. Culture on oatmeal-wheatgerm agar. C. Culture on double strength malt-yeast extract agar. D. Conidiomata from double strength malt-yeast extract agar (1 month). E-F. Conidiogenesis cells from oatmeal-wheatgerm agar. G-H. Conidia.


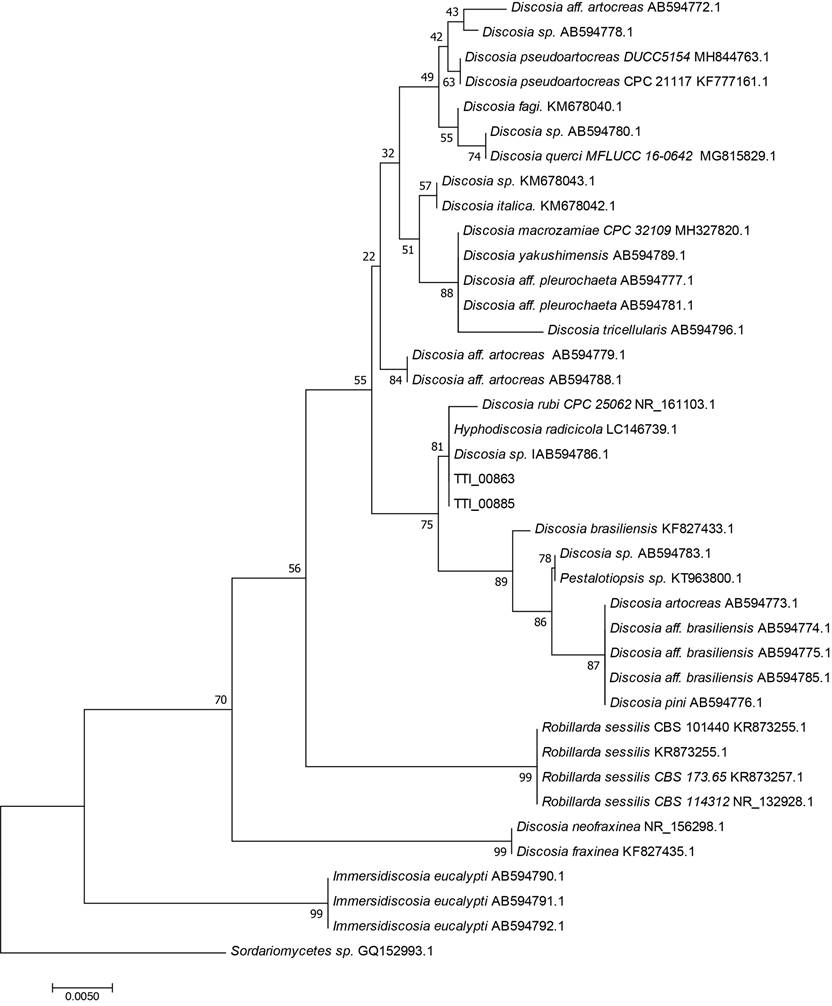


Figure S2. Approximate phylogenetic placement of strains TTI-0863 and TTI-0885 among fungi of the Sporocadaceae. Phylogenetic reconstruction was based on maximum likelihood analysis of an alignment of the ITS rDNA region. MEGA 7.0 under a K2+G model. Bootstrap support values >50% from 1000 replicates per run are labeled on branch nodes. Unidentified Sordariomycete species was designated the outgroup.


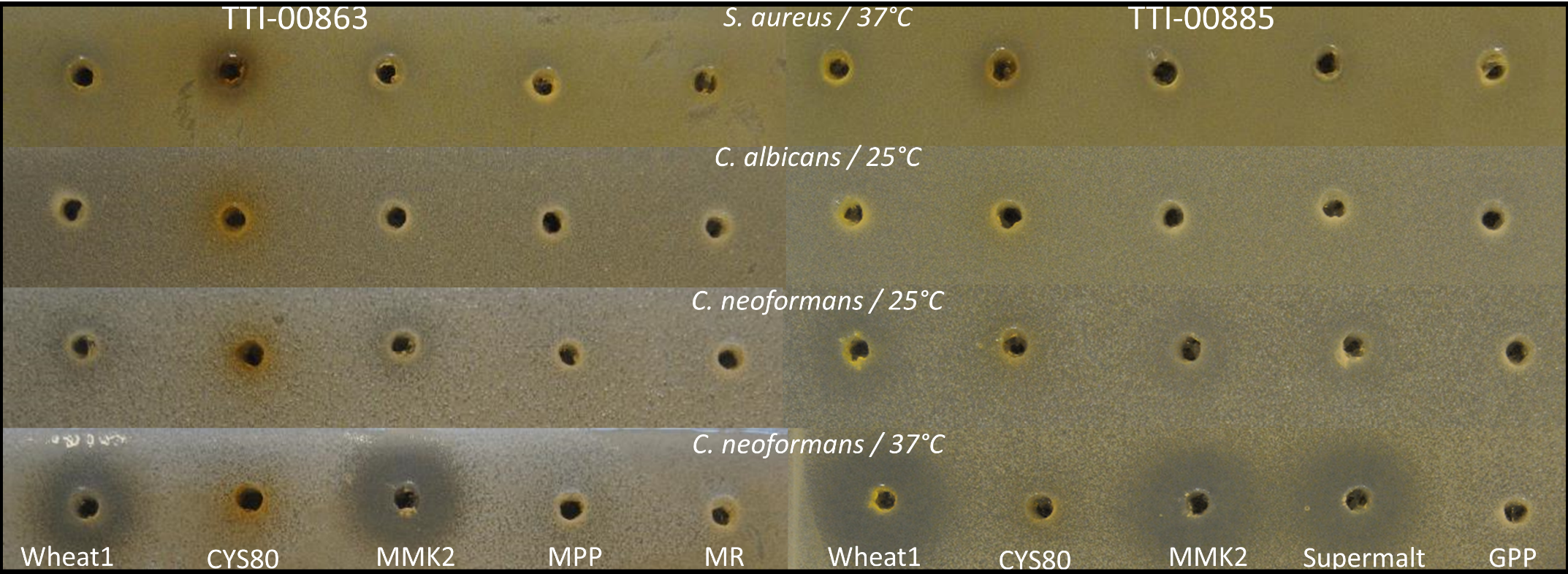


Figure S3. *Cryptococcus neoformans* agar inhibition zone assay using crude extracts of strains TTI-0863 (left half) and TTI-0885 (right half) showing temperature dependent anti-cryptococcal activity at 37 °C. Each strain was grown on five different media. Medium abbreviations are explained in Methods and Materials.


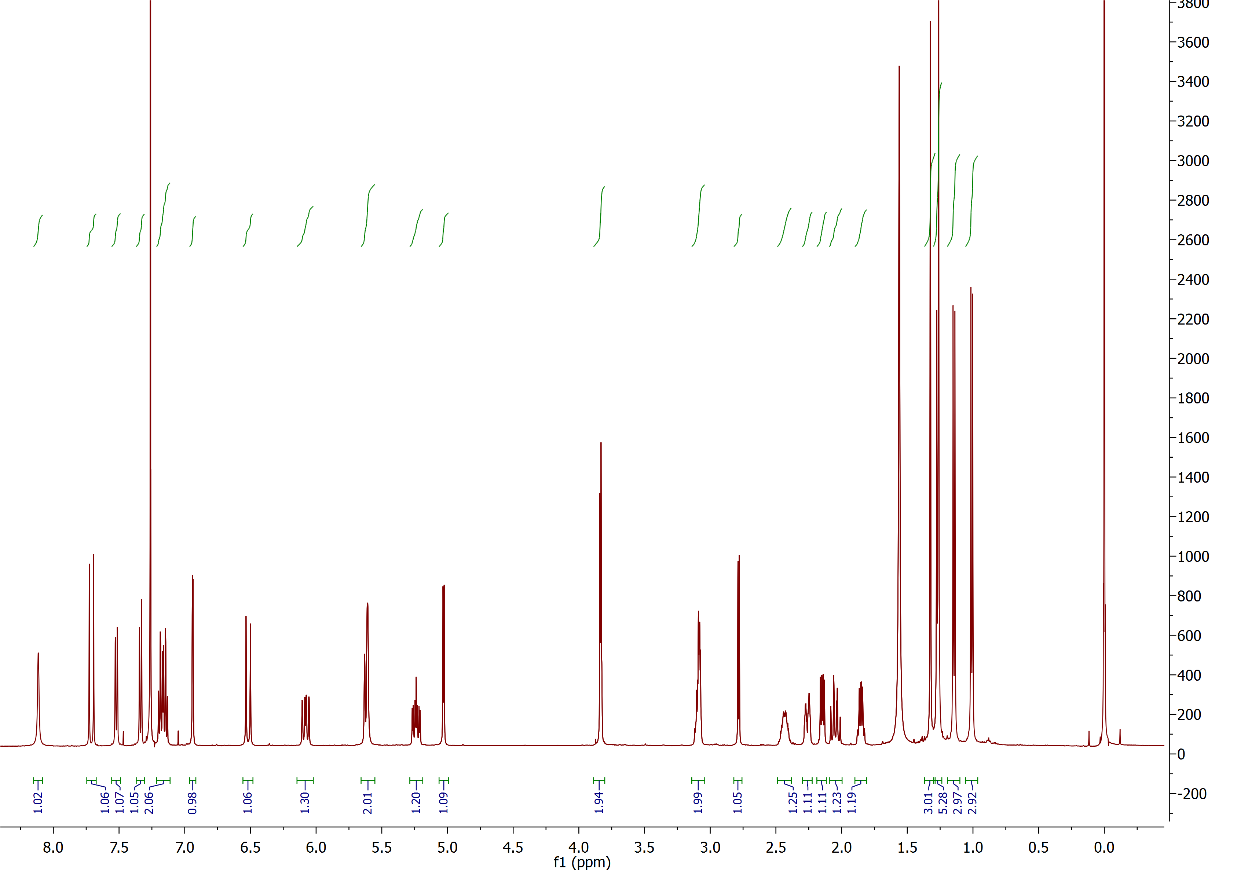


Figure S4. ^1^H spectrum of chaetoglobosin P (500MHz, CDCl_3_).


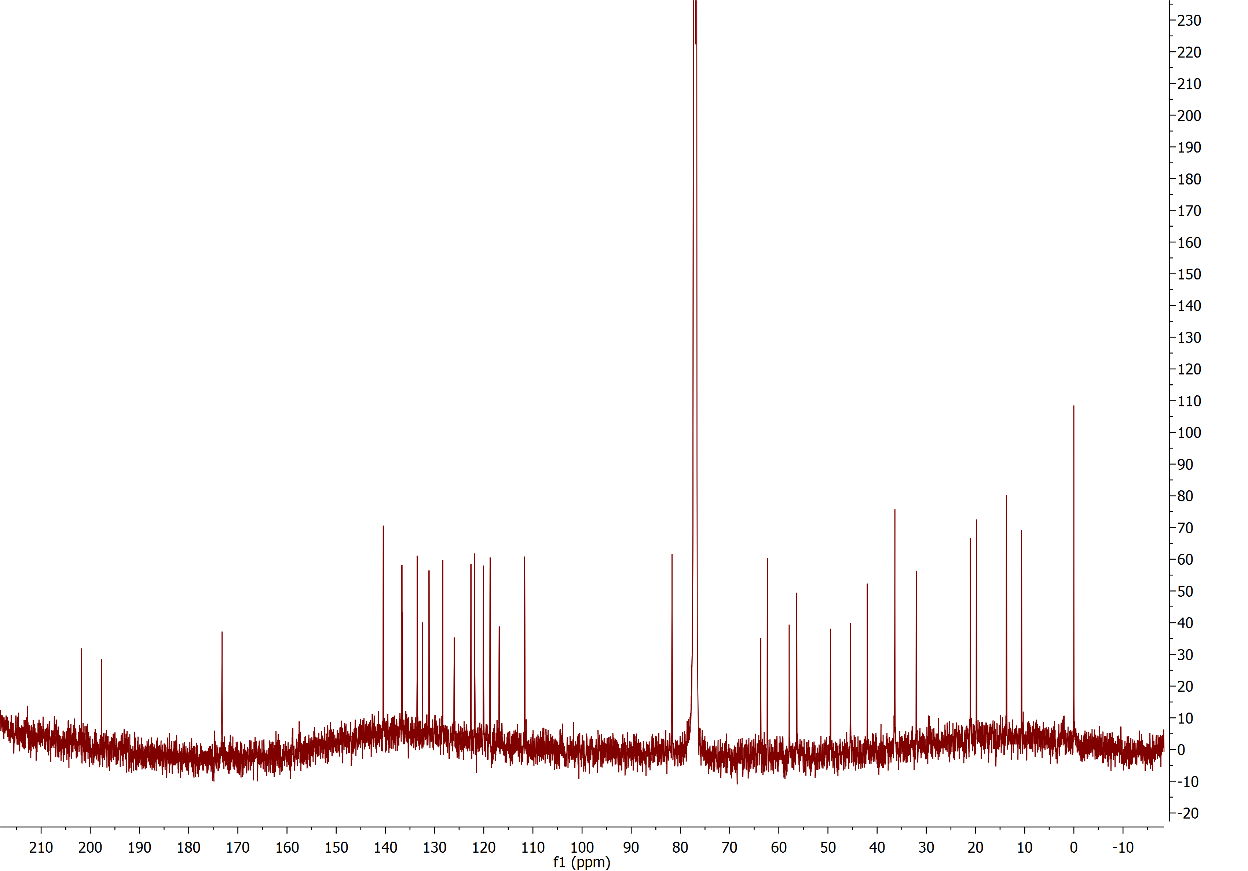


Figure S5. ^13^C spectrum of chaetoglobosin P (125 MHz, CDCl_3_).


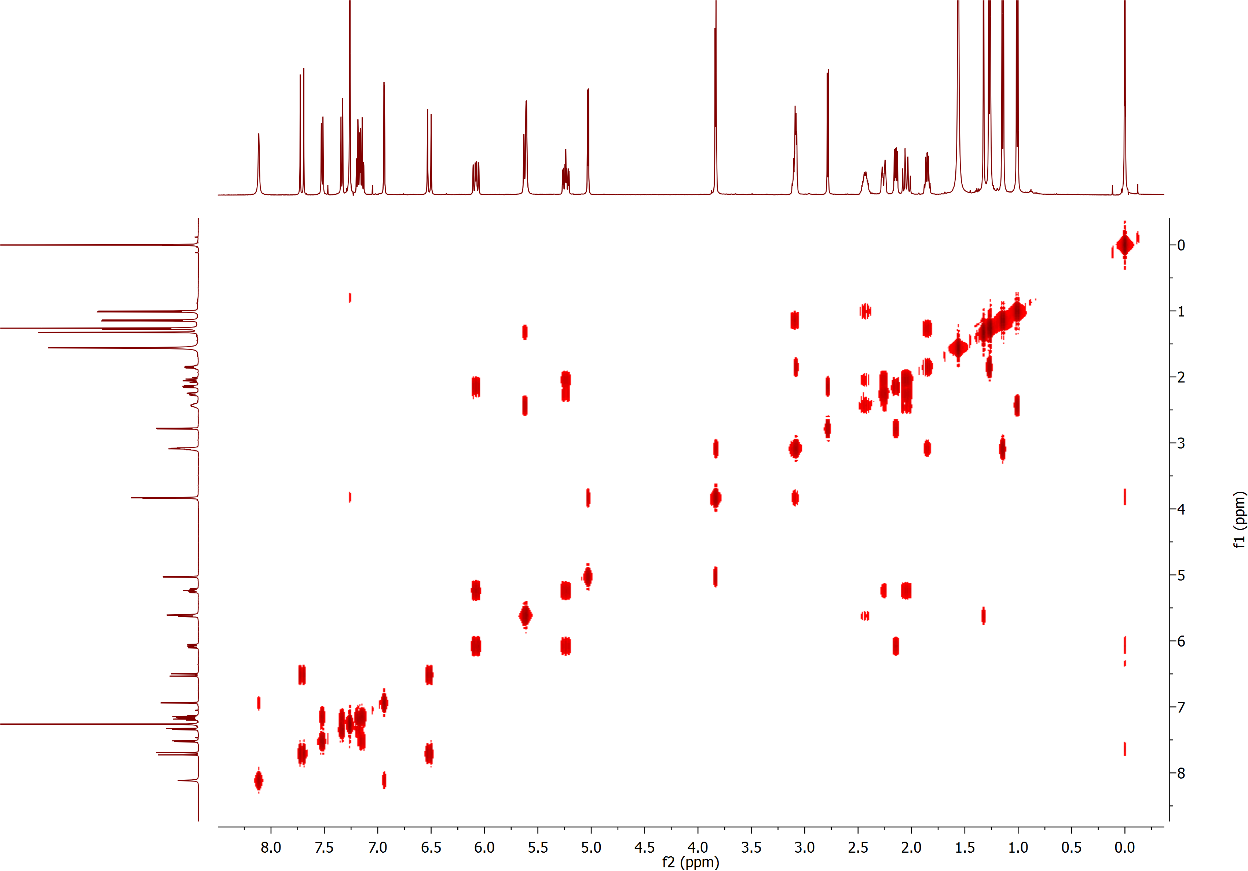


Figure S6. COSY spectrum of chaetoglobosin P (500 MHz, CDCl_3_).


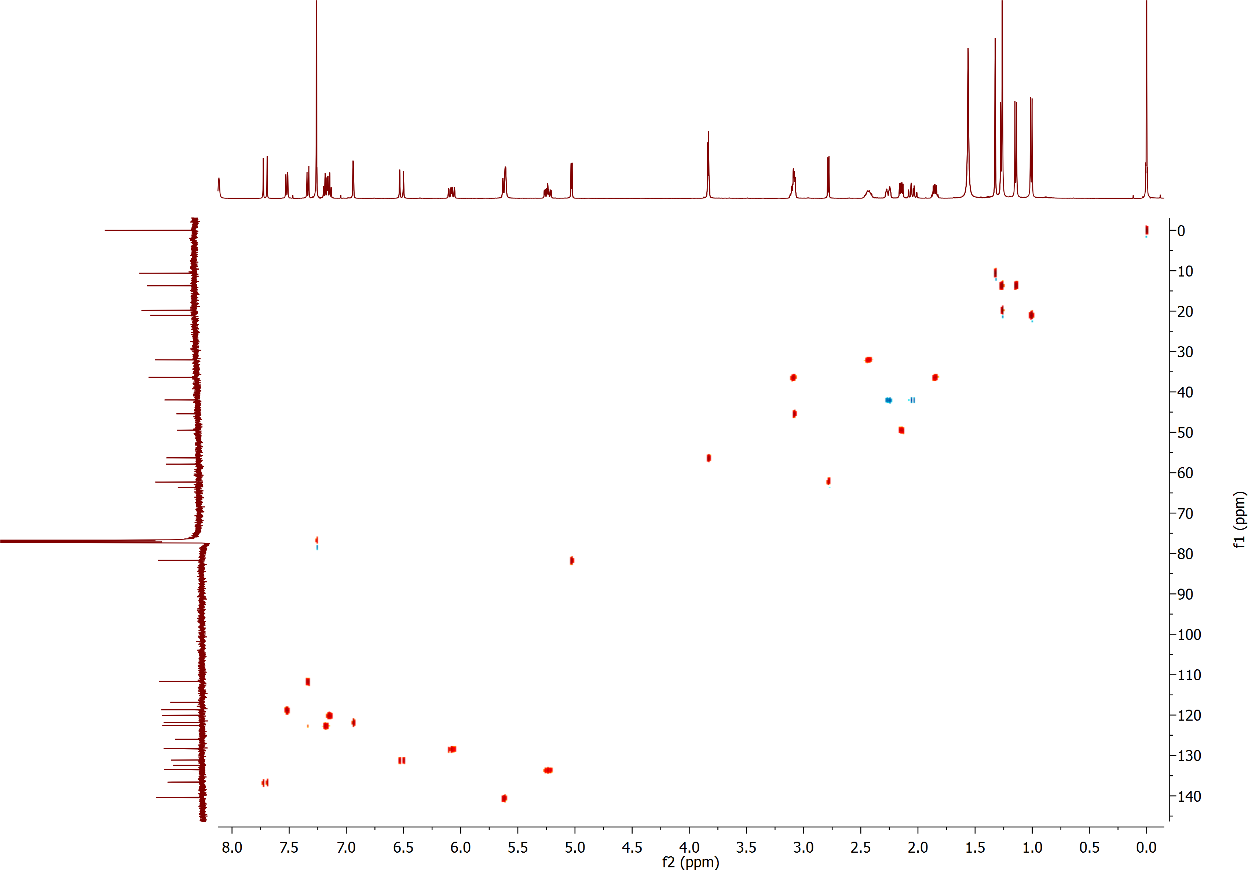


Figure S7. HSQC spectrum of chaetoglobosin P (500 MHz, CDCl_3_).


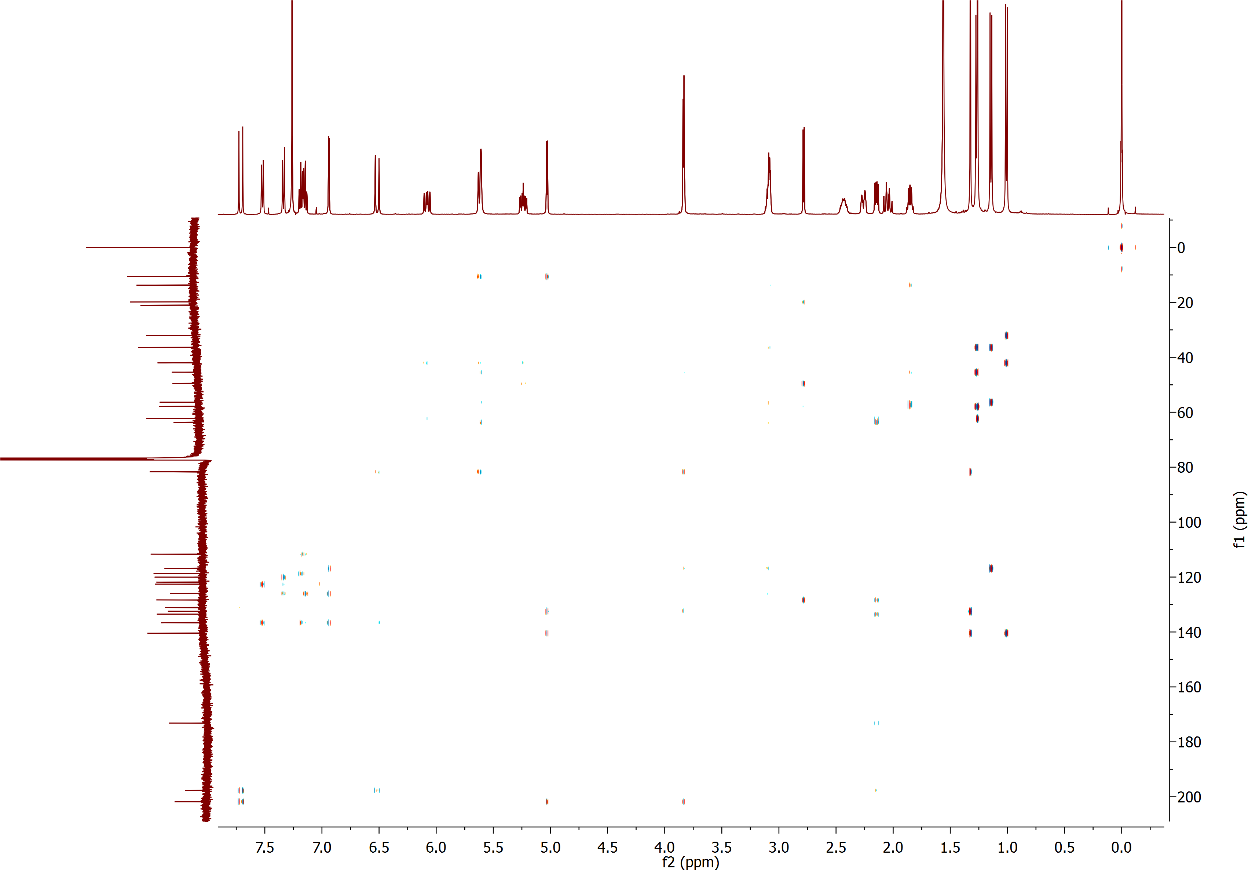


Figure S8. HMBC spectrum of chaetoglobosin P (500 MHz, CDCl_3_).


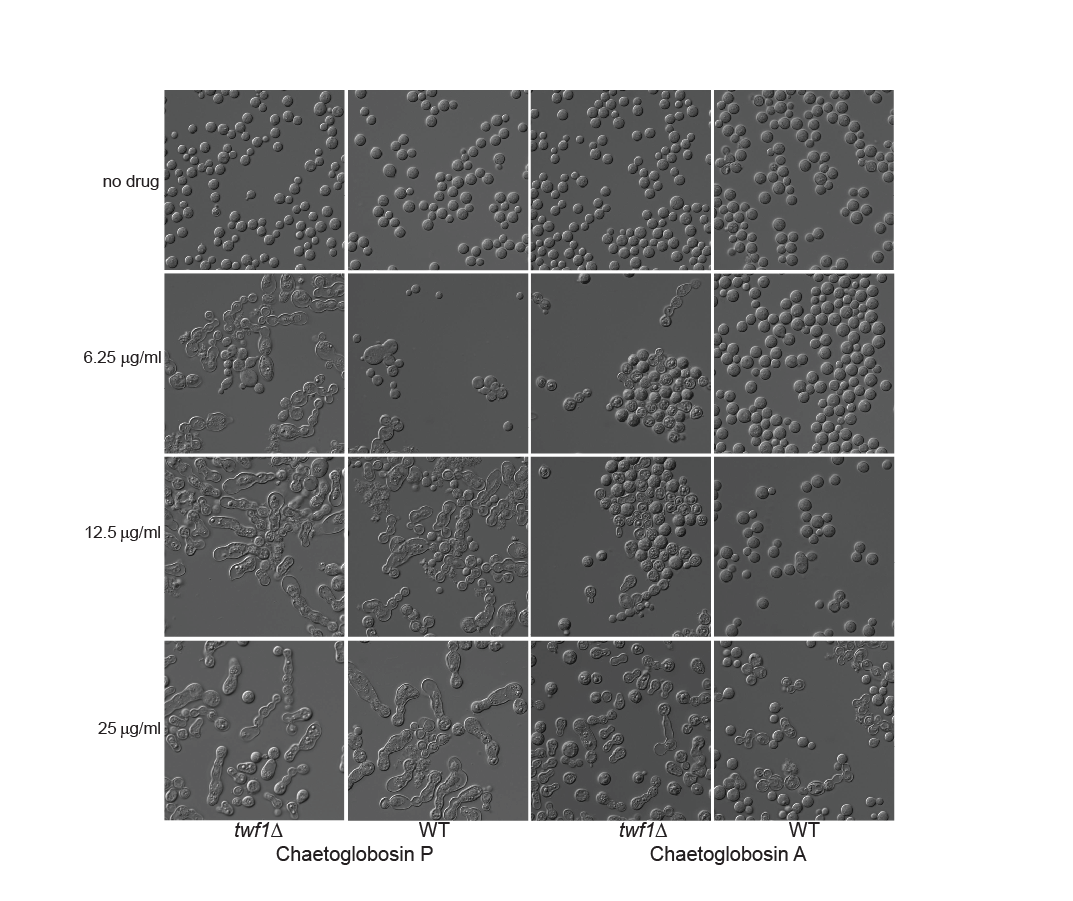


Figure S9. Effects of increasing concentrations of chaetoglobosins P and A on morphology of wild type (WT) and twinfilin-1 deletion (twf1Δ) mutants of *Cryptococcus neoformans*.

Table S1. List of mutants sensitive to chaetoglobosin P.

| **Strain** | **Gene ID** | **Gene name** | **Putative function** |
| --- | --- | --- | --- |
| sensitive | CNAG_03957 |  | 4-nitrophenyl phosphatase |
| sensitive | CNAG_04650 | Arp6 | actin-like protein arp6 |
| sensitive | CNAG_03622 | Mor2 | cell polarity protein mor2 |
| sensitive | CNAG_00546 | Chs4 | chitin synthase |
| sensitive | CNAG_01182 |  | cytoplasmic protein |
| sensitive | CNAG_02182 |  | D-lactaldehyde dehydrogenase |
| sensitive | CNAG_01422 |  | endoplasmic reticulum protein |
| sensitive | CNAG_06809 |  | IKS protein kinase |
| sensitive | CNAG_03266 |  | malate dehydrogenase, NAD-dependent |
| sensitive | CNAG_05584 |  | methylthioribulose-1-phosphate dehydratase |
| sensitive | CNAG_00897 |  | putative glucosidase |
| sensitive | CNAG_05742 | Stp1 | putative site-2 protease |
| sensitive | CNAG_02907 |  | tRNA (guanine-N(1)-)-methyltransferase |
| sensitive | CNAG_03322 |  | UDP-glucuronate decarboxylase |
| sensitive | CNAG_01722 | Vps13 | vacuolar protein sorting-associated protein |
|  |  |  |  |
| sensitive | CNAG_00532 |  | hypothetical protein |
| sensitive | CNAG_00583 |  | hypothetical protein |
| sensitive | CNAG_00588 |  | hypothetical protein |
| sensitive | CNAG_02910 |  | hypothetical protein |
| sensitive | CNAG_05064 |  | hypothetical protein |
| sensitive | CNAG_07441 |  | hypothetical protein |
| sensitive | CNAG_03417 |  | hypothetical protein |
| sensitive | CNAG_04363 |  | hypothetical protein |
| sensitive | CNAG_01514 |  | hypothetical protein |
| sensitive | CNAG_01825 |  | hypothetical protein |
| sensitive | CNAG_01931 |  | hypothetical protein |

Table S2. List of mutants resistant to chaetoglobosin P.

| **Strain** | **Gene ID** | **Gene name** | **Putative function** |
| --- | --- | --- | --- |
| resistant | CNAG_05921 |  | 2-acylglycerol O-acyltransferase 2 |
| resistant | CNAG_01400 | | 3-deoxy-7-phosphoheptulonate synthase |
| resistant | CNAG_04308 | | 3-hydroxyacyl-CoA dehydrogenase |
| resistant | CNAG_01076 | | 4-aminobutyrate aminotransferase |
| resistant | CNAG_03202 | Cac1 | Adenylate cyclase |
| resistant | CNAG_03452 | | AFG1 family mitochondrial ATPase |
| resistant | CNAG_00396 | Pka1 | AGC/PKA protein kinase |
| resistant | CNAG_00735 | | aldehyde dehydrogenase family 7 member A1 |
| resistant | CNAG_01257 | | aldo-keto reductase |
| resistant | CNAG_03413 | | alginate lyase |
| resistant | CNAG_03158 | Cmt1 | alpha-1,3-mannosyltransferase CMT1 |
| resistant | CNAG_05913 | | alpha-glucosidase |
| resistant | CNAG_04095 | | alpha-L-rhamnosidase |
| resistant | CNAG_00183 | Pcl12 | alternative cyclin Pcl12, variant |
| resistant | CNAG_00749 | | alternative sulfate transporter |
| resistant | CNAG_02365 | | Amidase |
| resistant | CNAG_03236 | | AMME syndrome candidate protein |
| resistant | CNAG_00235 | | amt family ammonium transporter |
| resistant | CNAG_06166 | | ATP-dependent DNA helicase MPH1 |
| resistant | CNAG_01340 | | ATPase |
| resistant | CNAG_03735 | | beta-1,2-xylosyltransferase |
| resistant | CNAG_06985 | | Beta-glucosidase |
| resistant | CNAG_01938 | Kin1 | CAMK/CAMKL/KIN1 protein kinase |
| resistant | CNAG_01156 | | capsular related protein |
| resistant | CNAG_00919 | | carboxypeptidase D |
| resistant | CNAG_02494 | | carboxypeptidase D |
| resistant | CNAG_05663 | Scw1 | cell wall integrity protein scw1 |
| resistant | CNAG_05734 | | cell wall organization and biogenesis-related protein, putative |
| resistant | CNAG_00799 | | cellulase |
| resistant | CNAG_02747 | | chaperone protein DNAJ |
| resistant | CNAG_06726 | Csr3 | chitin synthase regulator 3 |
| resistant | CNAG_04197 | | CMGC/DYRK/YAK protein kinase |
| resistant | CNAG_04627 | | COP9 signalosome complex subunit 12 |
| resistant | CNAG_05645 | | COP9 signalosome complex subunit 3 |
| resistant | CNAG_03083 | | cupin domain-containing protein |
| resistant | CNAG_02147 | | cytochrome c peroxidase |
| resistant | CNAG_05842 | | Cytochrome P450 |
| resistant | CNAG_02841 | | cytochrome P450 monooxygenase pc-2 |
| resistant | CNAG_00287 | | cytoplasmic protein |
| resistant | CNAG_02782 | | cytoplasmic protein |
| resistant | CNAG_07548 | | cytoplasmic protein |
| resistant | CNAG_02240 | | cytoplasmic protein |
| resistant | CNAG_05733 | | cytoplasmic protein |
| resistant | CNAG_07838 | | cytoplasmic protein |
| resistant | CNAG_06144 | | cytoplasmic protein |
| resistant | CNAG_06295 | | cytoplasmic protein, cytoplasmic protein, variant |
| resistant | CNAG_00029 | | D-3-phosphoglycerate dehydrogenase |
| resistant | CNAG_01540 | | dehydrogenase |
| resistant | CNAG_01044 | | dihydroceramidase |
| resistant | CNAG_03173 | | DNA damage-binding protein 1 |
| resistant | CNAG_07599 | PMS2 | DNA mismatch repair protein PMS2 |
| resistant | CNAG_05177 | | DNA polymerase kappa subunit |
| resistant | CNAG_01163 | | DNA repair and recombination protein RAD54-like protein |
| resistant | CNAG_07582 | | dynein light chain roadblock-type |
| resistant | CNAG_03101 | | efflux protein EncT |
| resistant | CNAG_04531 | | Enoyl-CoA hydratase |
| resistant | CNAG_00676 | Vps28 | ESCRT-I complex subunit VPS28 |
| resistant | CNAG_00876 | | ferric-chelate reductase |
| resistant | CNAG_01148 | | FK506-binding protein 4 |
| resistant | CNAG_01464 | | Flavohemoglobin |
| resistant | CNAG_04344 | | galactonate dehydratase |
| resistant | CNAG_01651 | | glicosidase |
| resistant | CNAG_02850 | | glucan endo-1,3-alpha-glucosidase agn1 |
| resistant | CNAG_06081 | | Glucose oxidase |
| resistant | CNAG_04350 | | glycerophosphoryl diester phosphodiesterase, glycerophosphoryl diester phosphodiesterase, variant |
| resistant | CNAG_05524 | | glycogen storage control protein |
| resistant | CNAG_04621 | | glycogen(starch) synthase, glycogen(starch) synthase, variant |
| resistant | CNAG_01080 | | Glycolate oxidase |
| resistant | CNAG_00407 | | glyoxal oxidase |
| resistant | CNAG_04505 | Gpa1 | guanine nucleotide-binding protein subunit alpha |
| resistant | CNAG_04470 | | Haloacid dehalogenase, type II |
| resistant | CNAG_07347 | | Heat shock protein |
| resistant | CNAG_02179 | | Hemolysin |
| resistant | CNAG_00315 | | HHE domain-containing protein |
| resistant | CNAG_05288 | | HUS1 checkpoint protein, HUS1 checkpoint protein, variant |
| resistant | CNAG_01029 | | impact family protein, impact family protein, variant |
| resistant | CNAG_05009 | | Integral membrane protein |
| resistant | CNAG_00257 | | kinesin family member 21A, variant |
| resistant | CNAG_05867 | | L-fucose transporter |
| resistant | CNAG_05461 | | L-lactate dehydrogenase (cytochrome) |
| resistant | CNAG_00865 | | maltose O-acetyltransferase |
| resistant | CNAG_01452 | | mat3 pheromone repeat protein |
| resistant | CNAG_06569 | | membrane transporter |
| resistant | CNAG_06497 | | microsomal epoxide hydrolase |
| resistant | CNAG_03403 | | mitochondrial protein |
| resistant | CNAG_04474 | | monocarboxylic acid transporter |
| resistant | CNAG_00874 | | multisite-specific tRNA:(cytosine-C5)- methyltransferase |
| resistant | CNAG_03910 | | myo-inositol transporter, putative |
| resistant | CNAG_02718 | | NAD binding dehydrogenase |
| resistant | CNAG_00094 | | NAD-dependent epimerase/dehydratase |
| resistant | CNAG_00149 | | NADH dehydrogenase (ubiquinone) 1 alpha subcomplex 4 |
| resistant | CNAG_05685 | | neutral amino acid transporter |
| resistant | CNAG_04343 | | nicotinamide mononucleotide permease |
| resistant | CNAG_02692 | | nitroreductase |
| resistant | CNAG_06544 | | non-histone chromosomal protein 6 |
| resistant | CNAG_02990 | | nuclear protein |
| resistant | CNAG_00411 | | nucleoporin p58/p45 |
| resistant | CNAG_03572 | | opsin 1 |
| resistant | CNAG_04112 | | oxidoreductase |
| resistant | CNAG_01377 | | PAB-dependent poly(A)-specific ribonuclease subunit PAN2 |
| resistant | CNAG_01172 | Pbx1 | parallel beta-helix repeat protein |
| resistant | CNAG_01652 | | peptide-N4-(N-acetyl-beta- glucosaminyl)asparagine amidase |
| resistant | CNAG_06837 | | PH domain-containing protein |
| resistant | CNAG_02104 | | phosphatidylinositol transfer protein SFH5 |
| resistant | CNAG_05677 | | phospholipase |
| resistant | CNAG_06967 | | Phytase |
| resistant | CNAG_05662 | | Polyol transporter protein 1 |
| resistant | CNAG_06347 | | pr4/barwin domain protein |
| resistant | CNAG_07903 | | Predicted Rho guanine nucleotide exchange factor (Rho GEF) |
| resistant | CNAG_02708 | | prenylcysteine oxidase/farnesylcysteine lyase |
| resistant | CNAG_06706 | | proline-, glutamic acid- and leucine-rich protein 1 |
| resistant | CNAG_04596 | | prolyl endopeptidase, prolyl endopeptidase, variant |
| resistant | CNAG_07804 | | protein mgr2 |
| resistant | CNAG_05155 | | protein-tyrosine-phosphatase |
| resistant | CNAG_01360 | | pumilio domain-containing protein c |
| resistant | CNAG_07701 | Ctr1 | putative copper ion transporter |
| resistant | CNAG_00125 | Crg1 | Putative regulator of G-protein signaling protein |
| resistant | CNAG_05785 | | putative transcription factor |
| resistant | CNAG_03719 | | Putative transporter of the major facilitator superfamily (MFS) |
| resistant | CNAG_02254 | | quinate permease |
| resistant | CNAG_04655 | | rab family protein |
| resistant | CNAG_02575 | | Rab small monomeric GTPase |
| resistant | CNAG_03722 | | RAN protein binding protein |
| resistant | CNAG_04761 | Ras2 | Ras family protein |
| resistant | CNAG_01672 | Ras/Rho | Ras family protein |
| resistant | CNAG_06593 | | rhamnogalacturonan lyase |
| resistant | CNAG_06606 | Rho104 | rho family GTPase |
| resistant | CNAG_04631 | | ribitol kinase |
| resistant | CNAG_04028 | | RNA binding protein, variant |
| resistant | CNAG_00036 | Sec14-2 | SEC14 cytosolic factor |
| resistant | CNAG_03153 | Sec14-1 | SEC14 cytosolic factor |
| resistant | CNAG_02584 | | serine/threonine-protein phosphatase 2A activator 1 |
| resistant | CNAG_05423 | | SET domain-containing protein |
| resistant | CNAG_03432 | | solute carrier family 2, solute carrier family 2, variant |
| resistant | CNAG_00654 | Rrx1 | sulfiredoxin |
| resistant | CNAG_01019 | Sod1 | superoxide dismutase [Cu-Zn] |
| resistant | CNAG_02187 | | terbinafine resistance locus protein |
| resistant | CNAG_02969 | | thioesterase, thioesterase, variant |
| resistant | CNAG_07866 | | transcription initiation factor TFIIA small subunit |
| resistant | CNAG_05934 | | translin domain protein |
| resistant | CNAG_00234 | | tRNA-dihydrouridine synthase 4 |
| resistant | CNAG_00106 | Tco5 | Two-component-like sensor kinase |
| resistant | CNAG_00363 | Tco6 | Two-component-like sensor kinase |
| resistant | CNAG_01850 | Tco1 | Two-component-like sensor kinase |
| resistant | CNAG_06278 | Tco7 | Two-component-like sensor kinase |
| resistant | CNAG_06920 | | ubiquitin carboxyl-terminal hydrolase 10 |
| resistant | CNAG_06637 | | ubiquitin carboxyl-terminal hydrolase 22/27/51 |
| resistant | CNAG_01084 | | ubiquitin-conjugating enzyme 4 |
| resistant | CNAG_03138 | | ubiquitin-protein ligase |
| resistant | CNAG_03133 | | UDP-glucose,sterol transferase |
| resistant | CNAG_04654 | | UNC-50 family protein |
| resistant | CNAG_00508 | Vps17 | vacuolar protein sorting-associated protein |
| resistant | CNAG_04306 | | vesicle transporter SFT2B |
| resistant | CNAG_05181 | | white collar 1 protein |
| resistant | CNAG_05835 | | wor1/pac2 family transcription factor |
| resistant | CNAG_00920 | | YjeF family protein |
| resistant | CNAG_07699 | | zinc finger family protein |
| resistant | CNAG_04697 | | zinc finger protein |
| resistant | CNAG_03964 | | zinc knuckle family protein |
| resistant | CNAG_00033 |  | hypothetical protein |
| resistant | CNAG_00049 | | hypothetical protein |
| resistant | CNAG_07313 | | hypothetical protein |
| resistant | CNAG_00127 | | hypothetical protein |
| resistant | CNAG_00129 | | hypothetical protein |
| resistant | CNAG_00177 | | hypothetical protein |
| resistant | CNAG_07339 | | hypothetical protein |
| resistant | CNAG_00242 | | hypothetical protein |
| resistant | CNAG_00275 | | hypothetical protein |
| resistant | CNAG_00276 | | hypothetical protein |
| resistant | CNAG_00277 | | hypothetical protein |
| resistant | CNAG_00279 | | hypothetical protein |
| resistant | CNAG_00302 | | hypothetical protein |
| resistant | CNAG_00369 | | hypothetical protein |
| resistant | CNAG_00391 | | hypothetical protein |
| resistant | CNAG_00446 | | hypothetical protein |
| resistant | CNAG_00476 | | hypothetical protein |
| resistant | CNAG_00487 | | hypothetical protein |
| resistant | CNAG_00568 | | hypothetical protein |
| resistant | CNAG_00663 | | hypothetical protein |
| resistant | CNAG_00690 | | hypothetical protein |
| resistant | CNAG_00739 | | hypothetical protein |
| resistant | CNAG_00766 | | hypothetical protein |
| resistant | CNAG_00841 | | hypothetical protein |
| resistant | CNAG_03741 | | hypothetical protein |
| resistant | CNAG_03752 | | hypothetical protein |
| resistant | CNAG_03755 | | hypothetical protein |
| resistant | CNAG_07486 | | hypothetical protein |
| resistant | CNAG_03974 | | hypothetical protein |
| resistant | CNAG_03978 | | hypothetical protein |
| resistant | CNAG_04059 | | hypothetical protein |
| resistant | CNAG_04064 | | hypothetical protein |
| resistant | CNAG_03054 | | hypothetical protein |
| resistant | CNAG_02905 | | hypothetical protein |
| resistant | CNAG_02878 | | hypothetical protein |
| resistant | CNAG_02864 | | hypothetical protein |
| resistant | CNAG_02845 | | hypothetical protein |
| resistant | CNAG_02788 | | hypothetical protein |
| resistant | CNAG_02719 | | hypothetical protein |
| resistant | CNAG_02685 | | hypothetical protein |
| resistant | CNAG_02667 | | hypothetical protein |
| resistant | CNAG_07566 | | hypothetical protein |
| resistant | CNAG_02596 | | hypothetical protein |
| resistant | CNAG_06904 | | hypothetical protein |
| resistant | CNAG_04934 | | hypothetical protein |
| resistant | CNAG_04974 | | hypothetical protein |
| resistant | CNAG_05226 | | hypothetical protein |
| resistant | CNAG_05227 | | hypothetical protein |
| resistant | CNAG_05255 | | hypothetical protein |
| resistant | CNAG_07827 | | hypothetical protein |
| resistant | CNAG_05325 | | hypothetical protein |
| resistant | CNAG_06867 | | hypothetical protein |
| resistant | CNAG_07415 | | hypothetical protein |
| resistant | CNAG_01417 | | hypothetical protein |
| resistant | CNAG_01249 | | hypothetical protein |
| resistant | CNAG_01228 | | hypothetical protein |
| resistant | CNAG_01223 | | hypothetical protein |
| resistant | CNAG_01180 | | hypothetical protein |
| resistant | CNAG_01171 | | hypothetical protein |
| resistant | CNAG_01154 | | hypothetical protein |
| resistant | CNAG_01089 | | hypothetical protein |
| resistant | CNAG_01028 | | hypothetical protein |
| resistant | CNAG_01010 | | hypothetical protein |
| resistant | CNAG_01008 | | hypothetical protein |
| resistant | CNAG_02540 | | hypothetical protein |
| resistant | CNAG_02441 | | hypothetical protein |
| resistant | CNAG_07631 | | hypothetical protein |
| resistant | CNAG_02393 | | hypothetical protein |
| resistant | CNAG_02381 | | hypothetical protein |
| resistant | CNAG_02188 | | hypothetical protein |
| resistant | CNAG_02176 | | hypothetical protein |
| resistant | CNAG_02093 | | hypothetical protein |
| resistant | CNAG_02041 | | hypothetical protein |
| resistant | CNAG_06647 | | hypothetical protein |
| resistant | CNAG_05772 | | hypothetical protein |
| resistant | CNAG_05889 | | hypothetical protein |
| resistant | CNAG_05915 | | hypothetical protein |
| resistant | CNAG_05940 | | hypothetical protein |
| resistant | CNAG_07698 | | hypothetical protein |
| resistant | CNAG_07702 | | hypothetical protein |
| resistant | CNAG_03085 | | hypothetical protein |
| resistant | CNAG_03135 | | hypothetical protein |
| resistant | CNAG_03328 | | hypothetical protein |
| resistant | CNAG_03382 | | hypothetical protein |
| resistant | CNAG_03416 | | hypothetical protein |
| resistant | CNAG_06993 | | hypothetical protein |
| resistant | CNAG_04106 | | hypothetical protein |
| resistant | CNAG_04167 | | hypothetical protein |
| resistant | CNAG_04184 | | hypothetical protein |
| resistant | CNAG_04227 | | hypothetical protein |
| resistant | CNAG_04254 | | hypothetical protein |
| resistant | CNAG_04317 | | hypothetical protein |
| resistant | CNAG_04321 | | hypothetical protein |
| resistant | CNAG_04327 | | hypothetical protein |
| resistant | CNAG_04526 | | hypothetical protein |
| resistant | CNAG_04847 | | hypothetical protein |
| resistant | CNAG_04607 | | hypothetical protein |
| resistant | CNAG_04592 | | hypothetical protein |
| resistant | CNAG_01501 | | hypothetical protein |
| resistant | CNAG_01506 | | hypothetical protein |
| resistant | CNAG_07593 | | hypothetical protein |
| resistant | CNAG_01835 | | hypothetical protein |
| resistant | CNAG_01855 | | hypothetical protein |
| resistant | CNAG_01857 | | hypothetical protein |
| resistant | CNAG_01982 | | hypothetical protein |
| resistant | CNAG_01993 | | hypothetical protein |
| resistant | CNAG_06067 | | hypothetical protein |
| resistant | CNAG_06214 | | hypothetical protein |
| resistant | CNAG_06289 | | hypothetical protein |
| resistant | CNAG_06297 | | hypothetical protein |
| resistant | CNAG_05452 | | hypothetical protein |
| resistant | CNAG_05639 | | hypothetical protein |
| resistant | CNAG_04212 | | hypothetical protein |
| resistant | CNAG_00317 | | hypothetical protein |
| resistant | CNAG_00780 | | hypothetical protein, hypothetical protein, variant |
| resistant | CNAG_02994 | | hypothetical protein, hypothetical protein, variant |
| resistant | CNAG_02934 | | hypothetical protein, hypothetical protein, variant |
| resistant | CNAG_06831 | | hypothetical protein, hypothetical protein, variant |
| resistant | CNAG_07458 | | hypothetical protein, hypothetical protein, variant |
| resistant | CNAG_02224 | | hypothetical protein, hypothetical protein, variant |
| resistant | CNAG_02143 | | hypothetical protein, hypothetical protein, variant |
| resistant | CNAG_06653 | | hypothetical protein, hypothetical protein, variant |
| resistant | CNAG_05784 | | hypothetical protein, hypothetical protein, variant |
| resistant | CNAG_03404 | | hypothetical protein, hypothetical protein, variant |
| resistant | CNAG_03455 | | hypothetical protein, hypothetical protein, variant |
| resistant | CNAG_04907 | | hypothetical protein, hypothetical protein, variant |
| resistant | CNAG_04587 | | hypothetical protein, hypothetical protein, variant |
| resistant | CNAG_01753 | | hypothetical protein, hypothetical protein, variant |
| resistant | CNAG_07601 | | hypothetical protein, hypothetical protein, variant |
| resistant | CNAG_06145 | | hypothetical protein, hypothetical protein, variant |
| resistant | CNAG_06357 | | hypothetical protein, hypothetical protein, variant |
| resistant | CNAG_05453 | | hypothetical protein, hypothetical protein, variant |
| resistant | CNAG_05501 | | hypothetical protein, hypothetical protein, variant |
| resistant | CNAG_05586 | | hypothetical protein, hypothetical protein, variant |
| resistant | CNAG_00024 | | hypothetical protein, hypothetical protein, variant |
